# Epigenetic silencing of *TBX1* inactivates the Hippo pathway to potentiate chemoresistance in esophageal squamous cell carcinoma

**DOI:** 10.1038/s41419-026-08934-4

**Published:** 2026-06-09

**Authors:** Zhong Xu, Gang Ma, Ying Shen, Li Tan, Xuejuan Sun, Lei Peng, Weiyan Peng, Lin Ye

**Affiliations:** 1 Department of Cardiothoracic Surgery, Chongqing Key Laboratory of Molecular Oncology and Epigenetics, The First Affiliated Hospital of Chongqing Medical University, Chongqing, China; 2 Department of Gastric Surgery, Tianjin Medical University Cancer Institute & Hospital, National Clinical Research Center for Cancer; Tianjin Key Laboratory of Digestive Cancer; Tianjin’s Clinical Research Center for Cancer, Tianjin, China; 3 Department of Endocrine and Breast Surgery, Chongqing Key Laboratory of Molecular Oncology and Epigenetics, The First Affiliated Hospital of Chongqing Medical University, Chongqing, China

**Keywords:** Chemotherapy, Tumour-suppressor proteins, Apoptosis

## Abstract

Neoadjuvant chemotherapy (NAC) is a key component of the standard treatment regimen for patients with advanced esophageal squamous cell carcinoma (ESCC); however, a substantial proportion of patients fail to benefit from NAC because of intrinsic or acquired chemoresistance, leading to disease progression. Aberrant promoter methylation of tumor suppressor genes has been implicated in the diminished chemotherapy response in patients with ESCC. In this study, we show that hypermethylation of the CpG island of the T-box Transcription Factor 1 (TBX1) promoter is frequently associated with nonresponse to NAC in patients with ESCC. Functional experiments indicate that TBX1 suppresses ESCC cell proliferation, induces apoptosis, and increases cisplatin sensitivity. Mechanistically, TBX1 activates the Hippo signaling pathway through two complementary mechanisms: transcriptional upregulation of MST1 and LATS1 and inhibition of F-actin polymerization. Restoration of TBX1 expression enhances chemosensitivity and reduces tumor growth in vivo. Collectively, our findings suggest that TBX1 promoter hypermethylation has the potential to serve as a predictive biomarker of the NAC response in ESCC and define an epigenetic mechanism in which TBX1 silencing contributes to chemoresistance through impairment of Hippo pathway activation. These results suggest that modulating TBX1 expression and Hippo pathway activity represents a potential therapeutic strategy for overcoming chemoresistance in ESCC.

**Figure:**
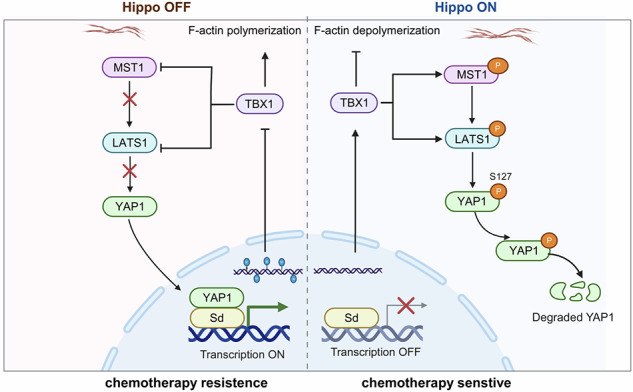

## Introduction

In 2022, 510,716 new cases of esophageal cancer (EC), which is the 11th most frequently diagnosed malignancy, and 445,129 deaths from EC, which is the seventh leading cause of cancer-related death, were reported globally [1]. Esophageal squamous cell carcinoma (ESCC) and esophageal adenocarcinoma are the two main histological subtypes, with ESCC being predominant [1]. Neoadjuvant chemotherapy (NAC) is important for patients with locally advanced ESCC, as it increases the resection rate, reduces recurrence, and ultimately improves patient survival outcomes [2, 3]. However, approximately 20% of tumors do not respond to NAC, and more than 50% exhibit only a partial response [4]. Accumulating evidence suggests that promoter methylation of tumor suppressor genes is significantly associated with reduced tumor sensitivity to NAC and consequently leads to poor prognosis in patients with EC [5, 6]. Thus, identifying epigenetic biomarkers and therapeutic targets relevant to the NAC response is of substantial clinical importance.

The T-box family of transcription factors plays essential roles in embryonic development and tumorigenesis. A structural hallmark of the protein family is the conserved T-domain, which mediates sequence-specific DNA binding [7]. TBX1 is indispensable for pharyngeal mesoderm development and is recognized as a key gene involved in 22q11.2 deletion syndrome [8, 9]. Accumulating evidence also indicates that TBX1 has tumor-suppressive effects on several malignancies, including thyroid cancer, murine skin tumors, and cervical cancer [10–12]. Notably, promoter methylation-mediated silencing of TBX1 has been shown to increase cisplatin resistance in cervical cancer [11]. However, the functional significance of TBX1 in ESCC has not yet been elucidated.

The Hippo signaling pathway is an evolutionarily conserved cascade signaling pathway that serves as a key regulator of cell proliferation, differentiation, survival, and organ homeostasis [13]. However, it is frequently dysregulated in cancer cells, and the expression of its transcriptional coactivator, YAP1, is closely associated with tumor initiation, progression, and chemoresistance [14–16]. In this pathway, the core effector YAP1 is negatively regulated by the upstream kinases MST1/2 and LATS1/2 [17]. Activation of MST1 requires homodimerization and autophosphorylation, followed by transphosphorylation of LATS1/2, which activates the Hippo pathway [18]. Moreover, loss of LATS1 expression has been shown to impair Hippo signaling [19]. As a transcriptional regulator of developmental processes, TBX1 functionally intersects with the Hippo pathway. Inactivation of Hippo signaling promotes the nuclear accumulation of YAP1, which is associated with poor prognosis in patients with EC [20]. In addition, the aberrant activation of YAP1 is closely linked to increased chemoresistance in part through the upregulation of EGFR expression in patients with EC [21]. Together, these findings raise the possibility that TBX1 may influence ESCC chemosensitivity by modulating Hippo signaling.

In this study, we report that the tumor-specific downregulation of TBX1 in patients who are resistant to NAC is mediated by promoter methylation. In addition, we demonstrated that TBX1 transcriptionally upregulates MST1 and LATS1, thereby activating Hippo signaling and promoting YAP1 degradation, leading to the suppression of ESCC cell proliferation and migration. Furthermore, restoration of TBX1 expression increased cisplatin sensitivity both in vitro and in vivo. Collectively, our findings suggest that TBX1 promoter methylation is a potential biomarker for predicting chemotherapy response and prognosis in ESCC patients, and support the therapeutic relevance of Hippo pathway activation in reducing tumor chemoresistance.

## Materials and methods

### Cell culture

The human esophageal squamous cell carcinoma lines KYSE150 and KYSE510 were obtained from the RIKEN BioResource Research Center (Ibaraki, Japan). Both cell lines were cultured in complete RPMI-1640 medium (Gibco, USA) supplemented with 10% fetal bovine serum (FBS; Biological Industries, Israel) and antibiotics (100 U/mL penicillin and 100 μg/mL streptomycin). The cells were incubated at 37 °C in a humidified 5% CO₂ atmosphere, and all the cells were mycoplasma-free.

### Generation of chemoresistant cells

Cisplatin-resistant KYSE150R and KYSE510R cells were generated by continuous exposure to gradually increasing concentrations of cisplatin (S1552; Beyotime, China). In brief, cells were seeded in a six-well plate at a density of 1 × 10^5^ cells/well. KYSE150 cells were treated with increasing concentrations of cisplatin (5–40 μM), and KYSE510 cells were treated with increasing concentrations of cisplatin (1–10 μM). The IC_50_ values were determined using a CCK-8 assay. Resistant cells were maintained in RPMI-1640 medium supplemented with 5 μM cisplatin (KYSE150R) or 1 μM cisplatin (KYSE510R).

### Tissue samples and evaluation of the response to NAC

Fresh ESCC tumor tissues were collected from patients who were classified as nonresponders to neoadjuvant chemotherapy (NR group) or those who achieved a partial response (PR group). All patients received docetaxel, cisplatin, and 5-fluorouracil-based chemotherapy without radiotherapy. Both male and female patients were included in this study. Sex was not considered a biological variable because the study was not designed to investigate sex-specific differences, and no sex-dependent effects were expected to influence the clinical outcomes. Additionally, paired fresh tumor and adjacent normal tissues were obtained from patients who underwent either esophagectomy or endoscopic biopsy. All the specimens were collected at the Department of Cardiothoracic Surgery, The First Affiliated Hospital of Chongqing Medical University. The relevant clinical characteristics of the patients are summarized in Tables 1 and 2.

**Table 1:** Clinicopathological features of TBX1 methylation in ESCC.

Clinicopathological features	Number(*n* = 48)
Gender	
Male	41(85.4%)
Female	7 (14.6%)
Age	
<65	26(54.2%)
≥65	22(45.8%)
Phase	
Ⅱ	19(39.6%)
Ⅲ	25(52.1%)
IV	4(8.3%)
Outcome	
alive	17(35.4%)
dead	31(64.6%)
Pathological classification	
squamous cell carcinoma	48(100%)
Response to Neoadjuvant Chemotherapy	
NR (non-responders)	26(54.2%)
PR (pathological complete response)	22(45.8%)

**Table 2:** Clinicopathological features of TBX1 expression in ESCC.

Clinicopathological features	Number(*n* = 16)
Gender	
Male	15(93.75%)
Female	1(6.25%)
Age	
<60	5(31.25%)
60–69	7(43.75%)
≥70	4(25%)
Phase	
Ⅰ	2(12.5%)
Ⅱ	5(31.25%)
Ⅲ	9(56.25%)
Outcome	
alive	10(62.5%)
dead	6(37.5%)
Pathological classification	
Squamous cell carcinoma	14(87.5%)
adenocarcinoma	2(12.5%)

The therapeutic response to NAC was evaluated according to the Response Evaluation Criteria in Solid Tumors (RECIST), version 1.1. Specifically, computed tomography (CT) images were analyzed using the following standards: a PR was defined as a ≥ 30% decrease in the sum of the longest diameters of target measurable lesions with no appearance of new lesions, whereas a NR was defined as a > 20% increase in the sum of the longest diameters, the development of new lesions, or stable disease.

This study was approved by the Institutional Ethics Committee of The First Affiliated Hospital of Chongqing Medical University and was conducted in accordance with the Declaration of Helsinki. Written informed consent was obtained from all the participating patients.

### Analyses using online databases and R software

To investigate the relationship between TBX1 promoter methylation and patient survival, we utilized the MethSurv database (biit.cs.ut.ee/methsurv/). The MethPrimer database (https://methprimer.com/) was used to design primers and analyze the methylation sites in the promoter region of the TBX1 gene. Analysis of the TBX1 promoter methylation status in EC tissues was performed using the UALCAN database (ualcan.path.uab.edu/). Potential transcription factor binding sites were predicted using the JASPAR database (https://jaspar.elixir.no/). GEO data (GSE104958) were used to analyze the relationships between TBX1 and YAP1 expression and resistance to platinum-based treatment regimens. For all analyses, a significance threshold of *p* < 0.05 was applied.

### Infinium methylation EPIC bead chip assay

Genome-wide DNA methylation profiling of tissue samples was performed using an Infinium Methylation EPIC Bead Chip array (850 K; Illumina, USA) according to the manufacturer’s standard protocol. Briefly, genomic DNA was bisulfite-converted, amplified, hybridized to the array, subjected to single-base extension, fluorescence staining, and scanned. Differential methylation analysis was performed at the CpG site level by comparing the β values between groups. CpG sites with an absolute Δβ ≥ 0.20 and a false discovery rate (FDR) < 0.05 were defined as differentially methylated CpG sites.

### Quantitative methylation-specific PCR

Bisulfite modification of DNA and BGS were carried out as described [22]. For qMSP, methylation-specific primers are described in Table 3. The qMSP primers are designed to target the sequence within the CpG island of the TBX1 gene promoter region in the GRCh38 genome version. This sequence encompasses two highly methylated CpG sites (cg02674728, cg06635590) showing relatively strong hypermethylation in the 850 K methylation array analysis of the TBX1 promoter.

**Table 3:** List of the sequences used in the study.

primer	Sense (5’ to 3’)	Anti-sense (5’ to 3’)
qMSP		
TBX1-m	GTGTTGTGGGTTAAGTATTTT	AAAACTCTACCCTCTCCTCCC
qPCR		
TBX1	GAGCCTTGGAAAGTTGTGAG	GGCATTTTCACACTACTGAAG
YAP1	TAGCCCTGCGTAGCCAGTTA	TCATGCTTAGTCCACTGTCTGT
STK4	AGTGCCAAAGGAGTGTCAATAC	GGATTCCTGGCGTTTCAGTTTC
LATS1	TTACCAAGATCCTCGACGAGAG	CACATTCCCTGGTTTCATGCT
β-actin	TCCTGTGGCATCCACGAAACT	GAAGCATTTGCGGTGGACGAT
GSN	GGTGTGGCATCAGGATTCAAG	TTTCATACCGATTGCTGTTGGA
CFL1	TACGCCACCTTTGTCAAGATG	CCTTGGAGCTGGCATAAATCAT
OCT4	CTTGAATCCCGAATGGAAAGGG	GTGTATATCCCAGGGTGATCCTC
Nanog	TTTGTGGGCCTGAAGAAAACT	AGGGCTGTCCTGAATAAGCAG
CD133	AGTCGGAAACTGGCAGATAGC	GGTAGTGTTGTACTGGGCCAAT
Chip		
MST1-1	GGGCTGATTGATCCTGAT	ATACGGATATTTGAAACCCCA
MST1-2	AGAACATTTTCATCACCCCAA	GATACGAGGGAAGGTGGTC
MST1-3	GGAGGTCTGTTTGGCATTGTG	GGACATTAGGGTCGTGAGACC
MST1-4	TCCTATTCATCAGCCAGGTGT	AACTCTGGTCGGACCCATTAT
MST1-5	TATGTCAGGGAATGGCTCCTT	CCGTATAGGAACCCTTTTCGT
LATS1-1	CCTTCTGCCTCAGCCTCC	AACGATACAATGGGTCCGACC
LATS1-2	TACCCAGGCTGGTCTTGAACT	GGTGGTTTGACCCGTCTTTCA
LATS1-3	CCTCAAGTGATCCGCCTGCCT	GTGTGGGCCGGTTATTTACAA
LATS1-4	TCCTGACCTCGTGATCCG	TGCCCAAAAGTGTAAACCTAAAG
LATS1-5	CCCAACGATCCCATCCCA	TGCCTACGCCAACTGTCT

### Plasmid transfection and generation of stable cell lines

The pcDNA3.1(+)-myc-TBX1 plasmid was constructed by BGI Tech Solutions Co., Ltd. (ShenZhen, China), and its sequence was verified. To generate pools of cells stably expressing TBX1, the full-length TBX1 expression plasmid was transfected into KYSE150R and KYSE510R cells using the Lipofectamine 2000 system (Invitrogen, Carlsbad, CA, USA). The cells were maintained in medium supplemented with G418 (ST081; Beyotime, China) for 14 days to establish stable cell lines, and the ectopic expression of TBX1 was confirmed by qRT-PCR and Western blotting. The stable cell lines were maintained in RPMI-1640 medium supplemented with 350 μg (KYSE150R) or 800 μg (KYSE510R) of G418 per milliliter.

### RNA extraction and qRT-PCR

Cell lines and tissue samples treated with DNase I were used for RNA extraction with TRIzol reagent (Invitrogen, USA). The RNA concentration was measured by spectrophotometry, and the storage temperature was −80 °C. RNA was reverse transcribed with a Promega Reverse Transcription System (Promega, Madison, WI, USA). qRT-PCR was performed using ABI SYBR Green Master Mix (RR820B; Takara, USA) in a QuantStudio 1 Plus Real-Time PCR System (Thermo Fisher Scientific, Waltham, Massachusetts, USA), and the thermal cycling conditions were as previously reported [23]. β-Actin was used as a loading control. The primers used are listed in Table 3.

### Western blot analysis and immunofluorescence staining

Western blot analysis: Proteins in total lysates extracted from cells were separated by sodium dodecyl sulfate-polyacrylamide gel electrophoresis (SDS-PAGE) and subsequently transferred onto polyvinylidene fluoride (PVDF) membranes (Millipore, USA). The membranes were then incubated with primary antibodies overnight at 4 °C prior to incubation with the appropriate horseradish peroxidase (HRP)-conjugated secondary antibodies. The protein bands were visualized using an enhanced chemiluminescence (ECL) detection system. Protein band intensities were quantified using ImageJ software. Relative expression levels were calculated by normalizing target protein signals to β-actin, and the value of the control group was set as 1.

Immunofluorescence (IF) Staining: Stably transfected cells and control cells were fixed with 4% paraformaldehyde (PFA) for 15 min at room temperature and permeabilized with 0.1% Triton X-100 for 10 min. Nonspecific binding sites were blocked by incubating the cells with 1% bovine serum albumin (BSA) in phosphate-buffered saline (PBS) for 1 h at room temperature. The cells were then incubated overnight at 4 °C with primary antibodies diluted in blocking buffer. Subsequently, the cells were washed and incubated with fluorophore-conjugated secondary antibodies for 1 h at room temperature in the dark. Nuclei were counterstained with DAPI (4’,6-diamidino-2-phenylindole) prior to imaging.

The primary antibodies used in this study were as follows: anti-Bax (#2772; CST, USA), anti-CDK4 (#12790; CST, USA), anti-Cyclin D1 (#2978; CST, USA), anti-Ki-67 (#9449; CST, USA), anti-Bcl2 (#3498; CST, USA), anti-TBX1 (bs-21501R;, Bioss, China), anti-Actin (sc-8432; Santa Cruz, USA), anti-STK3/STK4 (D261514; Sangon, China), anti-Caspase 3 (#9662; CST, USA), anti-CD133 (18470-1-AP; Proteintech, China), anti-OCT4 (TA422703; ORIGENE, China), anti-cleaved Caspase 3 (#9661; CST, USA), anti-LATS1/2(D151686; Sangon, China), anti-YAP1 (D194803; Sangon, China), anti-YAP1 phospho-Ser127 (D194803; Sangon, China), anti-phospho-MST1 (Thr 183)/MST2 (Thr 180) (F0855; Selleck, China), anti-phospho-LATS1 (Thr1079)/LATS2 (Thr1041) (HY-P81210; MedChemExpress, China), and anti-F-actin (C2201S; Beyotime, China).

### RNA sequencing

Total RNA was extracted from cells using TRIzol® reagent (Invitrogen, USA). RNA sequencing was conducted on a BGISEQ-500 system by BGI (Shenzhen, China). The experimental procedure followed the manufacturer’s instructions. Genes exhibiting differential expression were identified using the DESeq2 R/Bioconductor package, and genes whose |log2FC | > 1 and adjusted P value (FDR) < 0.05 were considered statistically significant. Follow-up analysis was performed using the Dr. Tom online analysis system.

### siRNA transfection

Predesigned siRNAs targeting TBX1 (HY-RS14270; MCE, China) were obtained from MedChemExpress (MCE). To perform TBX1 depletion, 5 × 10^5^ cells stably overexpressing TBX1 were transfected with siRNA oligo mix (50 nM) utilizing Lipofectamine 2000 reagent for 48 h according to the manufacturer’s protocol. The knockout efficiency was determined by qRT-PCR and Western blotting. The cells were then subjected to proliferation and apoptosis assays.

### Transwell assay

For cell migration, 2 × 10^5^ cells resuspended in 200 µl of serum-free medium were seeded in the upper chamber of uncoated Transwell chambers (8 µm pore size; Corning, USA), and 500 µl of medium supplemented with 10% FBS was added to the lower chamber. For invasion assays, we used 200 µg /ml Matrigel. After being incubated for 48 h, the cells that penetrated the lower surface of the membrane were fixed with 4% paraformaldehyde and stained with crystal violet. Finally, the cells in three randomly selected fields were counted. The experiment was performed in triplicate. Data analyses were performed with ImageJ software (NIH, USA).

### Cell viability and colony formation assays

Cell viability and proliferation were assessed using a Cell Counting Kit-8 (CCK-8) (C00412; Beyotime, China) assay and plate colony formation assays. For the CCK-8 assay, cells were seeded in 96-well plates at densities of 2000–5000 cells per well. Following the experimental treatments, 10 μl of CCK-8 solution was added to each well. The plates were incubated at 37 °C for 2 h, after which the absorbance was measured at 450 nm using a microplate reader. Prior to cell inoculation, the Hippo signaling pathway inhibitors XMU-MP-1 (A420532; Sangon, China), GA-017 (A418671; Sangon, China), CA-3 (S8661; Selleck, China) and Jasplakinolide (ab141409; Abcam, Cambridge, UK) were used for pretreatment according to the manufacturer’s protocol. For the colony formation assay, cells were plated in 6-well plates at low densities (100–400 cells per well). The culture medium was replaced every 3 days. After 14 days of incubation, the colonies were fixed with 4% paraformaldehyde and stained with 1.25% crystal violet solution. Colonies containing ≥50 cells were counted manually. All the experiments were performed with three biological replicates.

### Cell cycle and apoptosis assays

The effects of TBX1 on cell cycle progression and apoptosis were evaluated using flow cytometry. Cell cycle analysis: Stably transformed cells and control cells were harvested, fixed with 75% ethanol, and stored overnight at −20 °C. Fixed cells were subsequently resuspended in phosphate-buffered saline (PBS) and stained with a solution containing propidium iodide (PI; 30 μg/ml) and RNase A (40 μg/ml). After the cells were incubated for 30 min in the dark, the cell cycle distribution was analyzed using a FACSCalibur flow cytometer (BD Biosciences, San Jose, CA, USA). Apoptosis assay: cells were transiently transfected with 4 μg of either the pcDNA3.1-myc-TBX1 expression plasmid or the empty vector control. Forty-eight hours post-transfection, apoptosis was quantified by double staining with Annexin V-FITC and propidium iodide (PI) according to the manufacturer’s protocol. Apoptotic cells were detected using a FACSCalibur flow cytometer (BD Biosciences, San Jose, CA, USA), and the data were analyzed with FlowJo software V8.0 (BD Biosciences, San Jose, CA, USA).

### Luciferase reporter assay

One microgram of the pGL3 vector expressing either the MST1/LATS1 promoter, 1 μg of pCDH-TBX1, and 10 ng of the Renilla luciferase reporter vector were cotransfected into KYSE150R and KYSE510R cells in triplicate using Lipofectamine 3000. Luciferase activity was measured 48 h later using a Dual-Luciferase Reporter Assay System (Promega, USA). Firefly luciferase signal values were normalized to the control Renilla luciferase signal values, and the data are presented as the average of three experiments.

### Chromatin immunoprecipitation (ChIP) assay

A ChIP kit was purchased from Cell Signaling Technology (#56383; CST, USA), and the assay was performed according to the protocol. In brief, KYSE150R and KYSE510R cells were grown to 80–90% confluence in 100-mm dishes. Approximately 6×10^6^ cells per dish were subjected to crosslinking with 1% formaldehyde in PBS for 10 min at room temperature. The crosslinking reaction was quenched by adding 125 mM glycine for 5 min. The cells were then washed twice with ice-cold PBS, harvested by scraping, and pelleted by centrifugation. The cell pellets were resuspended in 1 mL of ice-cold ChIP Sonication Cell Lysis Buffer and incubated on ice for 10 min. After centrifugation, the nuclei were lysed in 1 mL of ChIP Sonication Nuclear Lysis Buffer containing protease inhibitors. Chromatin was sheared by sonication into fragments with an average size of 200–1000 bp by using a Branson sonifier. The sonicated lysates were clarified by centrifugation, and the supernatant was collected as the soluble chromatin fraction. For each immunoprecipitation reaction, 5–10 μg of sonicated chromatin was diluted with ChIP buffer and incubated overnight at 4 °C with the indicated specific antibody or normal IgG, IgG as a negative immunoprecipitation control. Protein G magnetic beads were then added, and the mixture was incubated for 2 h. The bead complexes were sequentially washed with low-salt and high-salt wash buffers. Bound chromatin was eluted with ChIP Elution Buffer, and crosslinking was reversed by adding NaCl and proteinase K and incubating at 65 °C for 2 h. DNA was subsequently purified using spin columns. The enrichment of specific genomic regions was analyzed through qPCR by using the primers listed in Table 3. All qPCRs were performed in triplicate, and the values were normalized to the input DNA. The data from three independent experiments are presented as the percentage of input (mean ± SD).

### Animals

Female BALB/c nude mice (5–6 weeks old, 16–20 g) were purchased from Beijing Vital River Laboratory Animal Technology Co., Ltd., and housed in the Animal Center of Chongqing Medical University. Only female mice were used in this study to minimize biological variability. Sex was not considered a biological variable because the experimental design was not intended to investigate sex-dependent effects.

To investigate the effect of TBX1 on tumorigenesis, the mice were randomly divided into two experimental groups (*n* = 10 mice per group). A priori power analysis using G*Power indicated that a sample size of *n* = 5 per group was sufficient to detect an effect size of 1.2 SD with 80% power at a significance level of 0.05. One mouse in the KYSE510R tumor model died accidentally and was excluded; no other animals were excluded, and all remaining mice completed the planned procedures. The mice in one group were injected subcutaneously in the right posterior flank with KYSE150R or KYSE510R cells overexpressing TBX1, whereas the mice in the control group were injected with empty vector control cells. Starting on day 7 postinjection, five randomly selected mice from each group began receiving intraperitoneal injections of cisplatin (5 mg/kg). The tumor volume was determined every three days using the formula (length × width²)/2. On day 15, the mice that did not receive cisplatin were euthanized for tumor assessment. On day 33, all the cisplatin-treated mice were euthanized, and the tumors were collected for further analysis. All tumor measurements were performed independently by researchers blinded to group allocation to minimize observational bias.

The mice were maintained on a 12-h light/dark cycle with ad libitum access to food and water. All animal maintenance, experimental, and euthanasia procedures complied with national and institutional ethical standards. The study protocol was approved by the Animal Use and Care Committee of the Chongqing Medical University Animal Center, and all the experiments were performed by qualified personnel. In this experiment, the maximum diameter of all tumors was less than 15 mm, and their maximum volume was less than 1000 mm^3^, in compliance with the requirements of the ethics committee.

### Quantitative and statistical analyses

Statistical analyses were conducted with GraphPad Prism 9.0 (GraphPad, USA). Normality of the data was assessed using the Shapiro-Wilk test. Levene’s test confirmed equal variances. According to the data distribution and comparison design, appropriate parametric or non-parametric tests were applied. Comparisons between two groups were performed using Student’s *t* test or the Mann-Whitney U test, as appropriate. Comparisons among multiple groups were performed using one-way or two-way analysis of variance (ANOVA) followed by the appropriate multiple-comparison test. Survival analysis utilized Kaplan-Meier survival curves. The measurement data are presented as the mean ± SD, and *P* < 0.05 was considered to indicate a significant difference.

## Results

### *TBX1* promoter hypermethylation is more frequent in ESCC tissues

**Fig. 1:**
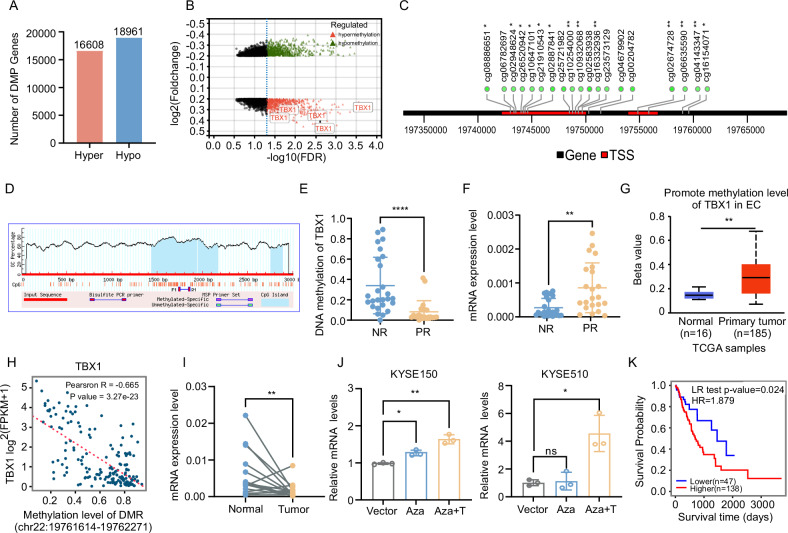
*TBX1* promoter hypermethylation is more frequent in ESCC tissues. **A** The numbers of hypomethylated and hypermethylated genes in esophageal tumor tissue samples from patients in the chemotherapy nonresponse (NR) and partial response (PR) groups, as determined by the Infinium HumanMethylation850 Bead Chip assay. **B** Volcano plot showing hypo- and hypermethylated gene loci identified by the Infinium HumanMethylation850 Bead Chip assay. **C** Differentially methylated probes within the TBX1 promoter region, with the green dots indicating a significantly hypermethylated site. **D** Two CpG islands in the TBX1 promoter region predicted using MethPrimer. **E** TBX1 promoter methylation status in esophageal tumor tissues from the NR (*n* = 26) and PR (*n* = 22) groups, as determined by qMSP (*p* < 0.0001). **F** Differential TBX1 mRNA expression in tissue samples in the NR and PR groups, as determined by qRT-PCR (*p* = 0.0013). **G** Predicted differential methylation of the TBX1 promoter between cancer and adjacent tissues, as determined using UALCAN. **H** Correlations between TBX1 mRNA expression and promoter methylation based on the analysis of methylation data. **I** Differential TBX1 mRNA expression between esophageal cancer and adjacent tissue samples, as determined by qRT-PCR (*p* = 0.0058). **J** Treatment with 5-aza-2′-deoxycytidine (5-Aza) or a combination of 5-Aza and trichostatin A (TSA) restored TBX1 mRNA expression in KYSE150 and KYSE510 cells, as determined by qRT-PCR analysis. **K** Association between TBX1 promoter methylation and patient prognosis in esophageal cancer, as determined using MethSurv (*p* = 0.024). (Data are presented as the mean ± SD values; ns, not significant; *p < 0.05; **p < 0.01.).

To investigate how DNA methylation contributes to chemoresistance mechanisms in ESCC, tumor tissues were collected from 48 patients receiving/undergoing NAC only and were further categorized into nonresponse (NR, *n* = 26) and partial response (PR, *n* = 22) groups according to the RECIST 1.1 criteria. Genome-wide DNA methylation profiling was then conducted using the Infinium HumanMethylation850 Bead Chip in these specimens, identifying 16 608 densely hypermethylated CpG sites in NR tissues compared with their PR counterparts (Fig. 1A). Notably, DNA sequences spanning from No.19 742 500 to No.19 750 000 as well as from No.19 754 000 to No.19 757 000 were heavily methylated and were located within the *TBX1* genetic locus (Fig. 1B, C). MethPrimer predictions revealed that these sequences contained CpG islands within the *TBX1* promoter region, spanning positions from approximately 1.5–2.2 kb and 2.7–2.9 kb upstream of its transcription start site (TSS) (Fig. 1D). The results of quantitative methylation-specific PCR (qMSP) further confirmed that *TBX1* promoter methylation levels were markedly higher in NR samples than in PR samples (Fig. 1E). As promoter hypermethylation generally leads to transcriptional silencing, *TBX1* mRNA expression was subsequently examined in these 48 specimens and was markedly lower in the NR group than in the PR group (Fig. 1F).

Notably, *TBX1* promoter hypermethylation was not limited to clinical samples obtained after chemotherapy but was also detected in treatment-naïve ESCC tissues, as evidenced by the finding that the methylation levels in tumor tissues were significantly higher than those in normal tissues in the TCGA EC dataset (Fig. 1G). Similarly, we found that methylation of *TBX1* was negatively linked to its mRNA expression, and this finding was further validated in our in-house tissues (Fig. 1H, I). In addition, treatment with the whole-genome demethylating agents 5-aza-2’-deoxycytidine (5-Aza) and trichostatin A (TSA) restored *TBX1* expression in the KYSE150 and KYSE510 ESCC cell lines (Fig. 1J). Kaplan-Meier analysis (with MethSurv) revealed that patients with *TBX1* hypermethylation had significantly shorter overall survival times (Fig. 1K). Collectively, these findings indicate that *TBX1* promoter hypermethylation is closely linked to transcriptional silencing, chemoresistance, and poor prognosis in patients with ESCC.

### TBX1 suppresses malignant phenotypes in chemoresistant ESCC cells

**Fig. 2:**
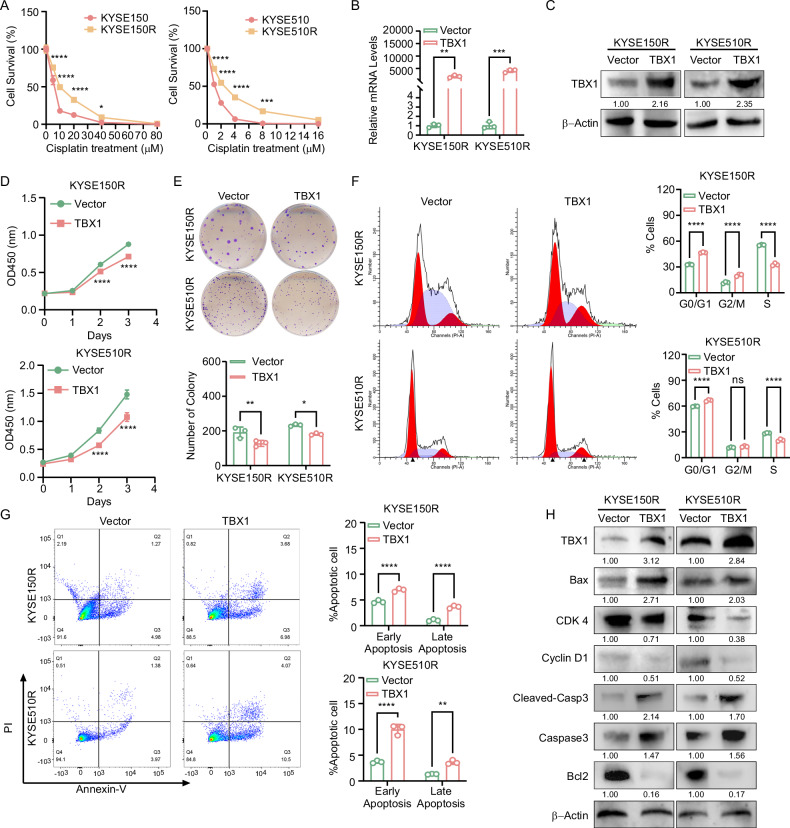
TBX1 suppresses ESCC cell proliferation and induces G_0_/G_1_ arrest and apoptosis in vitro. **A** Comparison between the responses of the naïve and cisplatin resistant KYSE150 and KYSE510 cell lines to cisplatin treatment. The IC_50_ values of cisplatin in the parental and resistant cell lines were 5.58 μM and 15.11 μM, respectively, for KYSE150R cells and 1.14 μM and 3.02 μM, respectively, for KYSE510R cells. **B** qRT-PCR analysis of TBX1 mRNA expression in stable transgenic cells after plasmid transfection. **C** Western blot analysis of TBX1 protein expression in stable transgenic cells after plasmid transfection. **D** CCK-8 assay results showing that TBX1 suppresses the proliferation of KYSE150R and KYSE510R cells. **E** Representative images and quantification of colony formation in control and TBX1-overexpressing cell lines. **F** Flow cytometric analysis results showing that TBX1 induces G_0_/G_1_ arrest in both KYSE150R and KYSE510R cells. **G** TBX1 promotes early apoptosis in KYSE150R and KYSE510R cells, as evaluated by flow cytometry. **H** Western blot results showing that in KYSE150R and KYSE510R cells, TBX1 overexpression decreased the expression of Cyclin D1, CDK4, and Bcl2 but increased the levels of Caspase-3, cleaved Caspase-3, and Bax. (Data are presented as the mean ± SD values; ns, not significant; *p < 0.05; **p < 0.01; ***p < 0.001; ****p < 0.0001.).

To clarify the functional role of TBX1 in chemoresistant ESCC cells, cisplatin-resistant ESCC cell sublines, namely KYSE150R and KYSE510R, were established. The half-maximal inhibitory concentration (IC_50_) of cisplatin was 15.11 μM in KYSE150R cells compared with 5.58 μM in parental KYSE150 cells, while the IC₅₀ was 3.02 μM in KYSE510R cells versus 1.14 μM in parental cells (Fig. 2A). TBX1 was subsequently overexpressed in both cisplatin-resistant cell lines (Fig. 2B, C). We found that TBX1 overexpression significantly inhibited the growth of both KYSE150R and KYSE510R cells by inducing G_0_/G_1_ arrest and consequently reducing the S-phase population (Fig. 2D–F). Both early and late apoptosis rates markedly increased following TBX1 overexpression (Fig. 2G). Moreover, overexpression of TBX1 downregulated Cyclin D1 and CDK4 expression and upregulated the expression of apoptosis-related proteins, including Caspase-3, cleaved Caspase-3, and Bax; and suppressed the antiapoptotic protein Bcl2 (Fig. 2H).

**Fig. 3:**
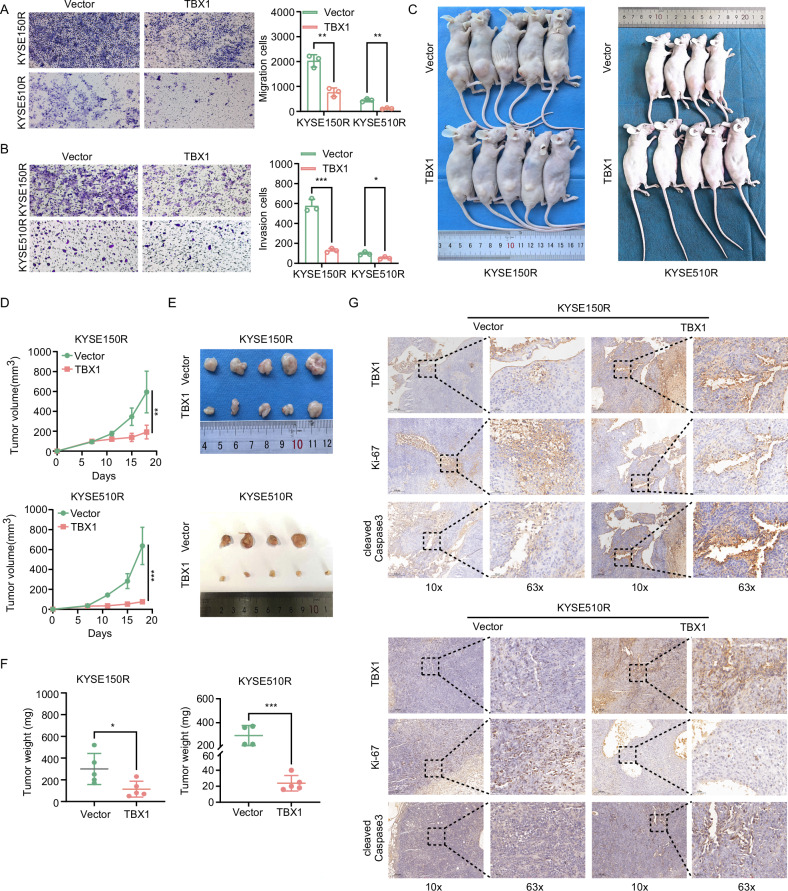
TBX1 overexpression suppresses migration, invasion, and tumor growth in cisplatin-resistant ESCC. **A** Representative images of Transwell migration studies with control and TBX1-overexpressing cells, showing a significant reduction in number of migrated cells in the TBX1-overexpressing groups of KYSE150R and KYSE510R cells. **B** Representative images of Transwell invasion studies with control and TBX1-overexpressing cells, showing a significant reduction in the number of invading cells in the TBX1-overexpressing groups of KYSE150R and KYSE510R cells. **C** Representative images of mouse xenograft models established with KYSE150R or KYSE510R cells transfected with empty vector or the TBX1 overexpression plasmid (*n* = 5 mice per group, except KYSE510R vector group *n* = 4; quantitative analyses used the actual sample sizes). **D** Tumor growth curve from the xenograft model established with KYSE150R (*p* = 0.038) or KYSE510R (*p* = 0.0003) cells transfected with empty vector or the TBX1 overexpression plasmid. **E** Ex vivo images of tumors isolated from the vector and TBX1-overexpressing groups of the KYSE150R or KYSE510R subcutaneous xenograft model. **F** Quantification of the weight of xenograft tumors isolated from different treatment groups. **G** Representative immunohistochemical image showing the expression of TBX1, Ki-67 and cleaved Caspase-3 in the xenograft tumor model. Scale bar, 200 μm. (Data are presented as the mean ± SD values; ns, not significant; *p < 0.05; **p < 0.01; ***p < 0.001; ****p < 0.0001.).

In addition, TBX1 restoration significantly inhibited the migration and invasion abilities of KYSE150R and KYSE510R cells (Fig. 3A, B). KYSE150R or KYSE510R cells with TBX1 overexpression and control counterparts were subcutaneously injected into nude mice, and the results revealed that TBX1 markedly inhibited tumor growth (Fig. 3C–F). Immunohistochemical (IHC) analysis revealed that compared with control tumors, TBX1-overexpressing tumors exhibited stronger TBX1 and cleaved Caspase-3 staining but markedly weaker Ki-67 staining, which was consistent with reduced proliferation and enhanced apoptosis. (Fig. 3G). These findings indicate that TBX1 mitigates the malignant phenotypes of chemoresistant ESCC cells both in vitro and in vivo.

### TBX1 enhances cisplatin sensitivity in ESCC cells

**Fig. 4:**
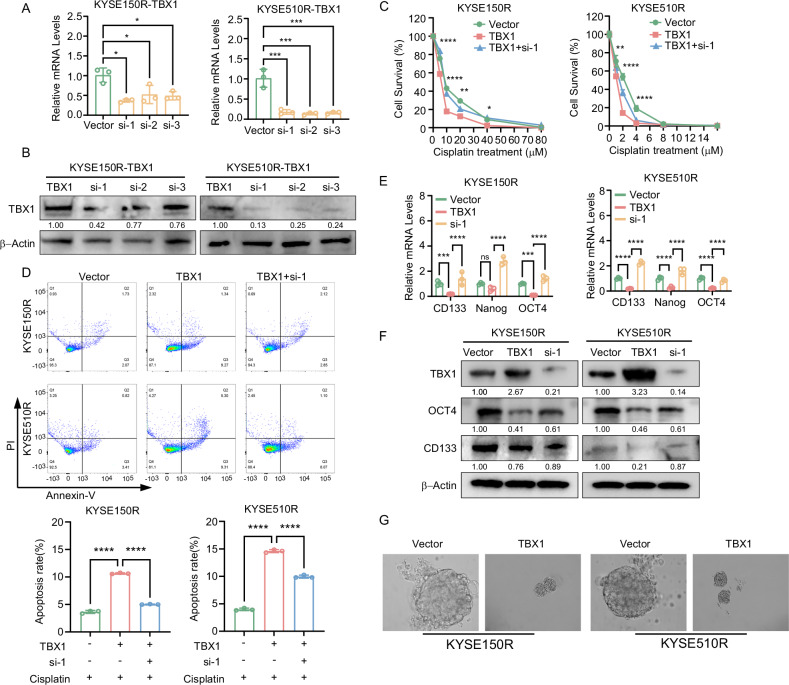
TBX1 enhances cisplatin sensitivity in ESCC cells in vitro. **A** qRT-PCR analysis of TBX1 mRNA expression in KYSE150R-TBX1 and KYSE510R-TBX1 cells stably transfected with siRNA. **B** Western blot analysis of TBX1 protein expression in KYSE150R-TBX1 and KYSE510R-TBX1 cells stably transfected with siRNA. **C** Overexpression of TBX1 sensitizes cisplatin-resistant KYSE150R and KYSE510R cells to cisplatin. The IC_50_ values of cisplatin in the vector-expressing and TBX1-overexpressing cells were 12.78 μM and 5.587 μM for KYSE150R cells and 2.646 μM and 0.934 μM, respectively, for KYSE510R cells and were restored to 11.12 μM in KYSE150R cells and 1.583 μM in KYSE510R cells after TBX1 knockdown. **D** Flow cytometric-based analysis showing that TBX1 increases cisplatin -induced apoptosis in KYSE150R (10 μM) and KYSE510R (2 μM) cells and that this effect is reversed by TBX1 knockdown. **E** Overexpression of TBX1 inhibited the expression of stemness-related markers (*CD133* and *OCT4*), as verified by qRT-PCR. **F** Western Blot showed that overexpression of TBX1 inhibited the expression of CD133 and OCT4. **G** Representative images of 3D sphere formation in the KYSE150R and KYSE510R cell lines showing spheroids formed per field of vision.

To further determine whether TBX1 loss undermines cisplatin sensitivity, siRNAs targeting TBX1 were used to knock down exogenous TBX1 in KYSE150R and KYSE510R cells. The siRNA with the highest knockdown efficiency (si-1) was selected for subsequent experiments (Fig. 4A, B). CCK-8 assays revealed that TBX1 overexpression significantly decreased the IC_50_ values, from 12.78 μM to 5.59 μM in KYSE150R cells and from 2.65 μM to 0.93 μM in KYSE510R cells; however, these effects were reversed by siRNA-mediated TBX1 knockdown, with which the IC₅₀ values increased to 11.12 μM and 1.58 μM, respectively (Fig. 4C).

Further analysis indicated that TBX1 overexpression enhanced cisplatin -induced apoptosis in both cell lines, whereas TBX1 knockdown significantly attenuated apoptosis following cisplatin treatment (Fig. 4D). Given that chemoresistant tumor cells often exhibit enhanced stemness [24], the expression of stemness markers was examined. TBX1 overexpression significantly decreased the mRNA and protein levels of *CD133* and *OCT4*, whereas these effects were reversed by TBX1 knockdown (Fig. 4E, F). Moreover, compared with the control, TBX1 overexpression significantly reduced the size of the tumor spheres derived from KYSE150R and KYSE510R cells, indicating that TBX1 could impair the self-renewal capacity of chemoresistant ESCC cells (Fig. 4G).

**Fig. 5:**
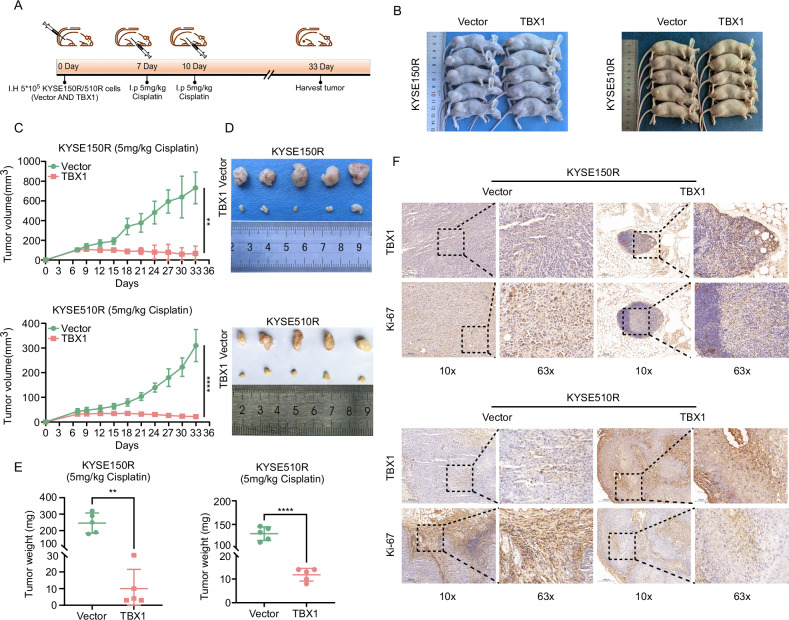
TBX1 enhances cisplatin sensitivity in ESCC cells in vivo. **A** KYSE150R or KYSE510R cells stably transfected with empty vector or TBX1 overexpression plasmid were subcutaneously injected into nude mice. After 7 days, cisplatin (5 mg/kg) was administered to the mice through intraperitoneal injection every three days throughout the indicated experimental period. **B** Representative image of xenograft tumors derived from KYSE150R or KYSE510R cells transfected with empty vector or the TBX1 overexpression plasmid following cisplatin treatment. **C** The tumor growth curve from the xenograft model established with KYSE150R (*p* = 0.0079) or KYSE510R (*p* < 0.0001) cells transfected with empty vector or the TBX1 overexpression plasmid after cisplatin treatment. **D** Ex vivo images of tumors isolated from the vector and TBX1 overexpression groups of the KYSE150R or KYSE510R subcutaneous xenograft model after cisplatin treatment. **E** Quantification of weight of xenograft tumors, isolated from different treatment groups. **F** Representative immunohistochemical image showing the expression of TBX1 and Ki-67 in the xenograft tumor model. Scale bar, 200 μm. (Data are presented as the mean ± SD values; ns, not significant; *p < 0.05; **p < 0.01; ***p < 0.001; ****p < 0.0001.).

To further validate the role of TBX1 in the response to cisplatin in vivo, murine xenograft models were established using KYSE150R and KYSE510R cells, both of them were treated with cisplatin (5 mg/kg) starting on day 7 (Fig. 5A). While tumors in the control group continued to grow under cisplatin treatment, TBX1-overexpressing tumors showed significantly suppressed growth, partial regression, and reduced tumor weight (Fig. 5B–E). Immunohistochemical (IHC) analysis revealed markedly weaker Ki-67 staining in TBX1-overexpressing tumors, which was consistent with reduced proliferative activity (Fig. 5F). Collectively, these results suggested that TBX1 enhances cisplatin sensitivity by promoting apoptosis and suppressing stemness in ESCC cells.

### TBX1 inhibits YAP1 activity in ESCC cells

**Fig. 6:**
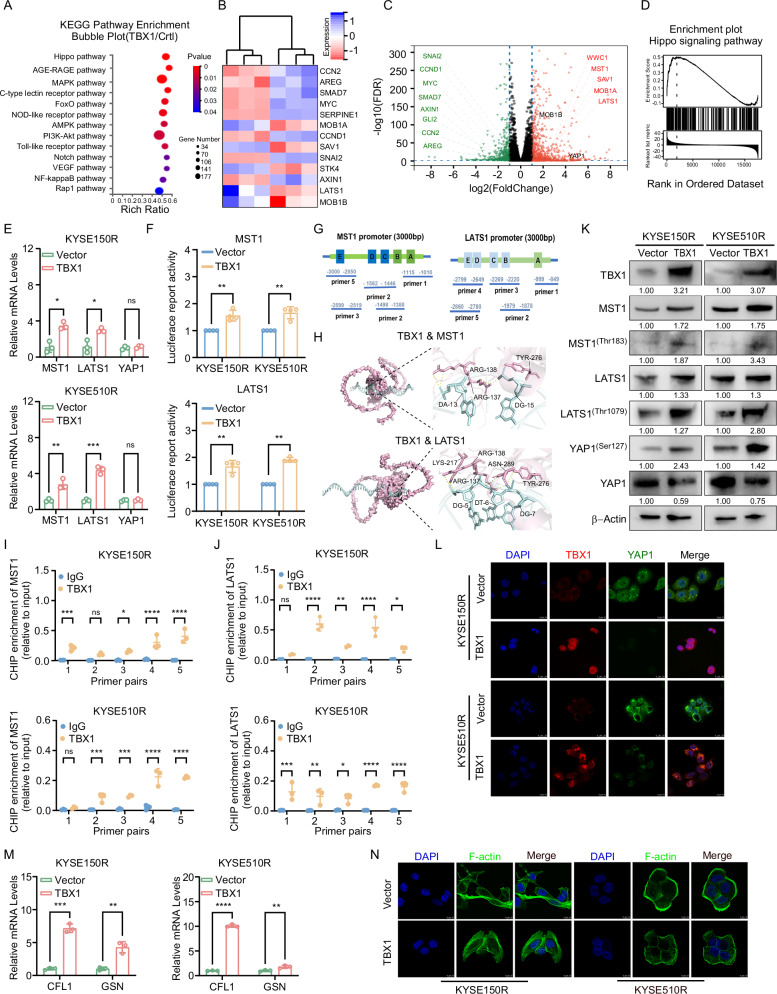
TBX1 inhibits YAP1 activity in ESCC cells. **A** Pathway analysis through gene set enrichment studies showing the pathways most strongly associated with the overexpression of TBX1 in KYSE150R cells. **B** Heatmap showing the changes in the expression of genes encoding proteins in the Hippo pathway according to the RNA-seq results. The heatmap displays the expression patterns of DEGs filtered by a false discovery rate (FDR) < 0.05. **C** Volcano plot of differentially expressed genes showing that *MST1* and *LATS1* are significantly upregulated by the overexpression of TBX1. Each dot represents one gene. Red dots indicate genes whose expression was significantly upregulated, and green dots indicate genes whose expression was downregulated on the basis of an FDR < 0.05 and a |log2FC | > 1. **D** GSEA revealed that TBX1 expression is positively associated with Hippo signaling pathway activation. **E** The regulatory effects of TBX1 on the expression of Hippo signaling pathway-related genes were verified by qRT-PCR. **F** Dual-luciferase reporter assay results confirming that TBX1 regulates the transcription and expression of *MST1* and *LATS1* in the KYSE150R and KYSE510R cell lines. **G** Potential TBX1-binding motifs in the *MST1* and *LATS1* promoters predicted by the JASPAR database. **H** PyMOL was used to construct TBX1-*MST1* and TBX1-*LATS1* promoter binding models for molecular docking simulation. **I** The binding of TBX1 to the *MST1* promoter was confirmed in both cell lines by ChIP-qPCR. **J** The binding of TBX1 to the *LATS1* promoter was confirmed in both cell lines by ChIP-qPCR, normal IgG was used as a negative control, and input chromatin was included to normalize the ChIP-qPCR data. **K** Western blot analysis results showing that TBX1 overexpression upregulated MST1 and LATS1 expression, increased YAP1 phosphorylation, and reduced total YAP1 protein levels. **L** Ectopic expression of TBX1 suppressed the nuclear translocation of YAP1 in KYSE150R and KYSE510R cells, as determined by immunofluorescence staining. **M** The regulatory effects of TBX1 on the expression of *CFL1* and *GSN* were verified by qRT-PCR. **N** Immunofluorescence staining results showing that ectopic expression of TBX1 inhibited F-actin polymerization and bundle formation in KYSE150R and KYSE510R cells. (Data are presented as the mean ± SD values; ns, not significant; *p < 0.05; **p < 0.01; ***p < 0.001; ****p < 0.0001.).

To investigate the mechanisms by which TBX1 attenuated chemotherapeutic activity, RNA sequencing was performed in the KYSE150R cell line. Differential expression analysis followed by KEGG enrichment analysis revealed significant enrichment of the Hippo signaling pathway (Fig. 6A). Volcano plots and heatmaps revealed that TBX1 overexpression increased the expression of upstream Hippo regulators but decreased the expression of downstream effectors (Fig. 6B, C). In addition, we performed gene set enrichment analysis (GSEA) using the TCGA dataset (EC) via LinkedOmics and further confirmed the strong association between TBX1 and Hippo signaling (Fig. 6D).

qRT-PCR analysis verified that *MST1* and *LATS1* expression was significantly upregulated in TBX1-overexpressing KYSE150R and KYSE510R cells (Fig. 6E). Dual-luciferase reporter assays revealed that TBX1 overexpression significantly increased the transcriptional activity of *MST1* and *LATS1* (Fig. 6F). The JASPAR database was used to predict the potential binding sites of TBX1 in the promoter regions of *MST1* and *LATS1* (Fig. 6G). Structural modeling using the AlphaFold Server further suggested that TBX1 could bind to upstream regulatory regions of the *MST1* and *LATS1* promoters (Fig. 6H). Chromatin immunoprecipitation (ChIP) assays confirmed that TBX1 directly binds to the promoters of *MST1* and *LATS1* (Fig. 6I, J). YAP1 function is paralyzed upon the phosphorylation of its Ser127 residue [25]. Western blot analysis revealed that TBX1 overexpression increased MST1, LATS1, p-MST1 (Thr183), and p-LATS1 (Thr1079) levels, thereby increasing YAP1 (Ser127) phosphorylation and degradation (Fig. 6K). Immunofluorescence staining further revealed that TBX1 overexpression significantly reduced nuclear YAP1 accumulation (Fig. 6L).

Previous reports have indicated that YAP1 activity is regulated by the conformation of the F-actin cytoskeleton. F-actin blockade/cleavage proteins, such as cofilin and gelsolin, are key gatekeepers that limit this regulatory mechanism, regulating YAP1 activity by modulating F-actin polymerization [26–30]. We examined the effect of TBX1 on actin remodeling. qRT-PCR revealed that TBX1 overexpression increased the transcription of cofilin (*CFL1*) and gelsolin (*GSN*) (Fig. 6M). Phalloidin staining revealed that TBX1 overexpression disrupted F-actin bundle formation (Fig. 6N). Collectively, these results indicate that TBX1 activated the Hippo signaling pathway through both transcriptional regulation of MST1/LATS1 and suppression of F-actin polymerization, thereby promoting YAP1 degradation and enhancing chemosensitivity. This dual regulation promotes YAP1 phosphorylation and degradation, thereby enhancing chemotherapy sensitivity.

### Hippo pathway activation is required for TBX1-mediated chemosensitivity in ESCC cells

**Fig. 7:**
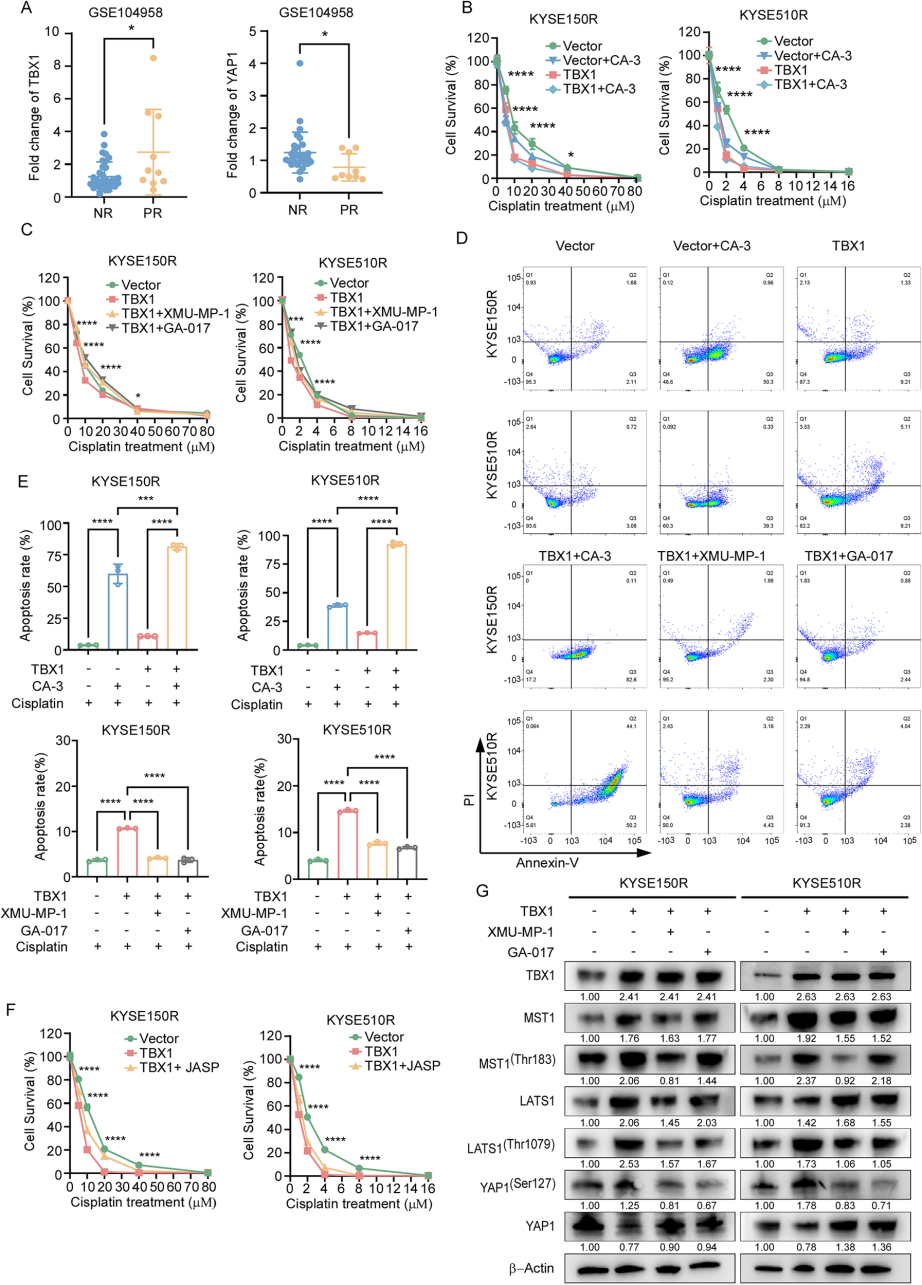
Hippo pathway activation is required for TBX1-mediated chemosensitivity in ESCC cells. **A** TBX1 and YAP1 expression in the EC patient dataset using GSE104958, including 9 PR patients and 31 NR patients who received neoadjuvant therapy with docetaxel, cisplatin, and 5-fluorouracil. **B** Inhibition of YAP1 by CA-3 (0.5 μM, 24 h) and overexpression of TBX1 increase the sensitivity of cisplatin-resistant KYSE150R and KYSE510R cells to cisplatin. The IC₅₀ values of cisplatin in the vector, vector/CA-3, TBX1, and TBX1/CA-3 groups were 12.78 μM, 5.728 μM, 5.587 μM, and 4.166 μM, respectively, for KYSE150R cells. The corresponding IC₅₀ values for the KYSE510R cells were 2.695 μM, 1.206 μM, 0.934 μM, and 0.5887 μM. **C** Pharmacological inhibition of the Hippo pathway attenuated the TBX1-mediated increase in cisplatin sensitivity in cisplatin-resistant KYSE150R and KYSE510R cells. Treatment with the Hippo pathway inhibitors XMU-MP-1 (1 μM, 24 h) or GA-017 (10 μM, 24 h) significantly reversed the increase in cisplatin sensitivity induced by TBX1 overexpression. Following XMU-MP-1 treatment, the IC₅₀ values of cisplatin were restored to 13.23 μM and 1.761 μM in KYSE150R and KYSE510R cells, respectively. Similarly, GA-017 treatment restored the IC₅₀ values to 14.34 μM and 2.075 μM in KYSE150R and KYSE510R cells, respectively. **D** Flow cytometry analysis showing that TBX1 overexpression significantly enhanced cisplatin-induced apoptosis in KYSE150R (10 μM) and KYSE510R (2 μM) cells. This proapoptotic effect was further potentiated by the YAP1 inhibitor CA-3 and markedly attenuated by treatment with the Hippo pathway inhibitors XMU-MP-1 or GA-017. **E** Quantification of apoptosis rates in the indicated treatment groups. **F** Pharmacologically induced F-actin polymerization can attenuate the TBX1-mediated increase in cisplatin sensitivity in cisplatin-resistant KYSE150R and KYSE510R cells. After treatment with Jasplakinolide (JASP, 0.5 μM, 1 h), the increase in cisplatin sensitivity induced by TBX1 overexpression was significantly reversed. The IC_50_ values of cisplatin in KYSE150R and KYSE510R cells were restored to 9.27 μM and 1.499 μM, respectively. **G** Western blot analysis showing that treatment with the Hippo pathway inhibitors XMU-MP-1 and GA-017 markedly reversed the TBX1-induced activation of the Hippo pathway and restored YAP1 expression. (Data are presented as the mean ± SD values; ns, not significant; *p < 0.05; **p < 0.01; ***p < 0.001; ****p < 0.0001.).

RNA-seq analysis of NAC-treated patient samples (NR = 31, PR = 9) proved that lower TBX1 expression and higher YAP1 expression were closely associated with platinum resistance (Fig. 7A). To determine whether the chemo-sensitizing effect of TBX1 on cisplatin treatment depends on Hippo pathway activation, we established ESCC cell models with TBX1 overexpression alone or in combination with YAP1 inhibition (CA-3) or Hippo pathway inhibition (XMU-MP-1 or GA-017) or promote F-actin polymerization (Jasplakinolide, JASP). YAP1 inhibition was applied to mimic the inhibition of YAP1 activity by Hippo pathway activation, whereas XMU-MP-1 and GA-017 were used to abolish MST1 and LATS1 phosphorylation, respectively. CCK-8 assays revealed that compared with control cells, TBX1-overexpressing cells or YAP1-inhibited cells displayed markedly reduced the IC₅₀ values to cisplatin, whereas inhibition of the Hippo pathway restored the IC₅₀ values to levels comparable to those of control cells (Fig. 7B, C). Consistently, flow cytometry further suggested that either YAP1 inhibition or TBX1 overexpression increased cisplatin-induced apoptosis, whereas inhibition of Hippo signaling significantly attenuated TBX1-mediated apoptosis (Fig. 7D, E). Moreover, promoting F-actin cytoskeleton polymerization also rescued the TBX1-mediated chemosensitization effect, further reinforcing the proposed mechanistic link between F-actin dynamics and Hippo/YAP1 signaling (Fig. 7F). Western blot analysis further confirmed that XMU-MP-1 and GA-017 reversed TBX1-induced Hippo pathway activation and restored YAP1 expression (Fig. 7G). Together, these results indicate that Hippo pathway activation is required for TBX1-mediated chemosensitivity in ESCC cells.

## Discussion

EC has a high incidence rate, and its mortality rate is the seventh highest among all tumors [31]. Chemoresistance resulting in ineffective NAC is an important factor contributing to high mortality rates. NAC remains the standard treatment for locally advanced ESCC, but its therapeutic efficacy is limited, with nearly 70% of patients exhibiting partial or no response, resulting in poor survival outcomes [3]. Cisplatin is a platinum-based drug widely used in NAC, yet resistance to cisplatin remains a major obstacle [32, 33]. Thus, understanding the molecular mechanisms underlying cisplatin resistance and identifying therapeutic targets are essential for improving treatment response.

DNA methylation is increasingly recognized as a clinically relevant biomarker for cancer detection and therapy prediction. High-throughput sequencing has facilitated the identification of methylation profiles with diagnostic and prognostic potential [34, 35]. However, most reported markers lack sufficient validation and clinical translation [36]. In this study, we identified TBX1 promoter hypermethylation as a frequent event in ESCC tumors with chemoresistance. Although patients with stable disease (SD) and progressive disease (PD) were initially classified together as the non‑responder (NR) group, stratified analysis revealed that the difference in TBX1 methylation between SD and PD subgroups was not statistically significant, possibly owing to the limited sample size. Furthermore, we confirmed that TBX1 showed significant differential methylation between tumor tissues and adjacent normal tissues, and demethylating agent treatment restored TBX1 expression in ESCC cells. Notably, although the restoration of TBX1 expression is consistent with epigenetic regulation, both 5-Aza and TSA exert genome-wide effects. Therefore, these results should be interpreted as supportive evidence for epigenetic regulation of TBX1 expression rather than definitive proof of locus-specific regulation at the TBX1 promoter. Furthermore, TBX1 promoter hypermethylation was strongly correlated with poor patient prognosis. These findings suggest that TBX1 promoter methylation may serve as a predictive biomarker for cisplatin-based neoadjuvant therapy in ESCC, with potential application in patient stratification.

Functionally, our data establish TBX1 as a tumor suppressor that promotes G_0_/G_1_ arrest, increases apoptosis, and reduces the proliferation and stemness of ESCC cells, which is consistent with the functions of its family members TBX2, TBX5, and TBX15 [37–39]. These effects contribute to increased chemosensitivity both in vitro and in vivo. Reduced apoptosis and enhanced stemness are recognized drivers of chemoresistance [40–42], which is consistent with our observation that TBX1 silencing diminished cisplatin sensitivity. These results provide the first evidence that TBX1 downregulation exerts pro-oncogenic effects on ESCC cells through epigenetic silencing.

Mechanistically, we indicated that TBX1 activates the Hippo signaling pathway through two complementary mechanisms: transcriptional upregulation of MST1 and LATS1, and inhibition of F-actin polymerization. This dual regulatory mechanism promotes YAP1 phosphorylation and degradation, reduces nuclear translocation, and ultimately increases cisplatin sensitivity. These results are consistent with those of previous studies that linked Hippo pathway inactivation and YAP1 activation to ESCC progression and drug resistance [13, 43–48]. Our findings suggest that TBX1 is an upstream regulator of Hippo signaling in ESCC cells. Moreover, inhibition of Hippo pathway activity abrogated the chemosensitizing effects of TBX1 and attenuated cisplatin-induced apoptosis, underscoring the functional importance of this pathway. Pharmacological inhibition with XMU-MP-1, GA-017, and CA-3 further supported the involvement of Hippo signaling in TBX1-mediated regulation. However, because small-molecule inhibitors may have off-target effects, these data should be interpreted as complementary evidence rather than definitive proof of pathway-specific causality. Interestingly, given the role of F-actin dynamics in mechanotransduction and Hippo pathway regulation [49, 50], TBX1 may also participate in mechanoresponsive signaling that influences drug resistance, a hypothesis that warrants future exploration.

Although this study reveals the dual mechanisms by which TBX1 transcriptionally activates the Hippo pathway and suppresses F-actin polymerization, which together promote YAP1 degradation, several limitations warrant future investigation. While our validation tests using clinical samples provide strong support, larger-scale, multicenter prospective cohort studies are essential to confirm the clinical value of TBX1 methylation as a predictive biomarker. Furthermore, owing to budget and time constraints, we were unable to establish a gene-knockout mouse model to study TBX1 function under cisplatin-resistant conditions in vivo, which limited our ability to discern the dynamic role of TBX1 in overcoming drug resistance. Additionally, the specific mechanism by which TBX1 regulates actin cytoskeleton polymerization remains incompletely explored. This study focused on TBX1 silencing caused by promoter methylation. However, whether other mechanisms, such as copy number variations, posttranscriptional regulation, or alterations in protein stability, contribute to TBX1 inactivation remains unclear. Although we confirmed that YAP1 inactivation is a key event, the specific downstream effectors of the Hippo pathway that directly mediate cell cycle arrest, apoptosis induction, and chemosensitization following TBX1 activation require further elucidation.

## Supplementary information

original data

Original Western blots

## Data Availability

The datasets used and/or analysed during the current study are available from the corresponding author on reasonable request.
