# Supplementary material for: Epigenetic silencing of TBX1 inactivates the Hippo pathway to potentiate chemoresistance in esophageal squamous cell carcinoma
Source: Cell Death Dis. 2026 Jun 9;17(1):727. doi: 10.1038/s41419-026-08934-4 (PMC13473094; doi:10.1038/s41419-026-08934-4)

Full unedited gel for Figure 2C

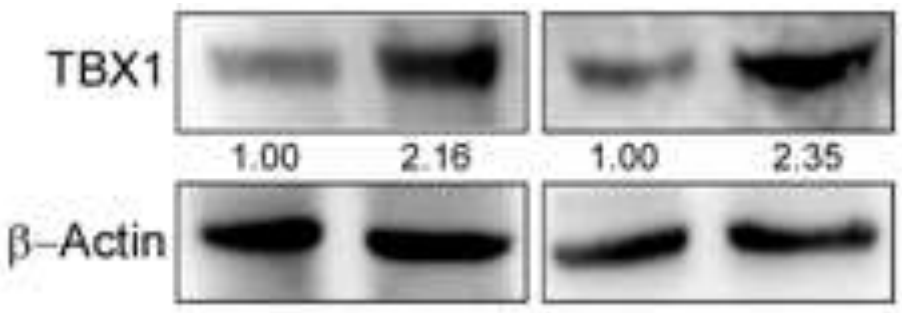

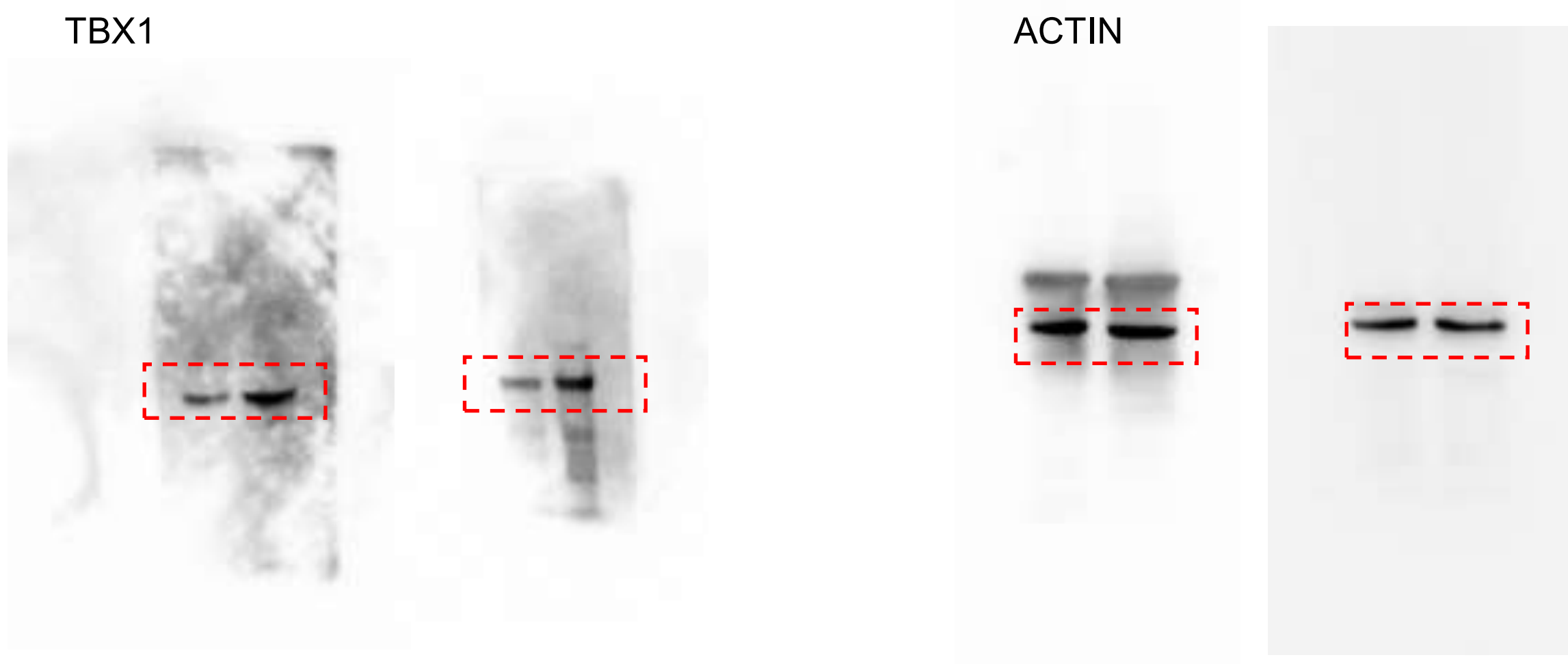

Full unedited gel for Figure 2H

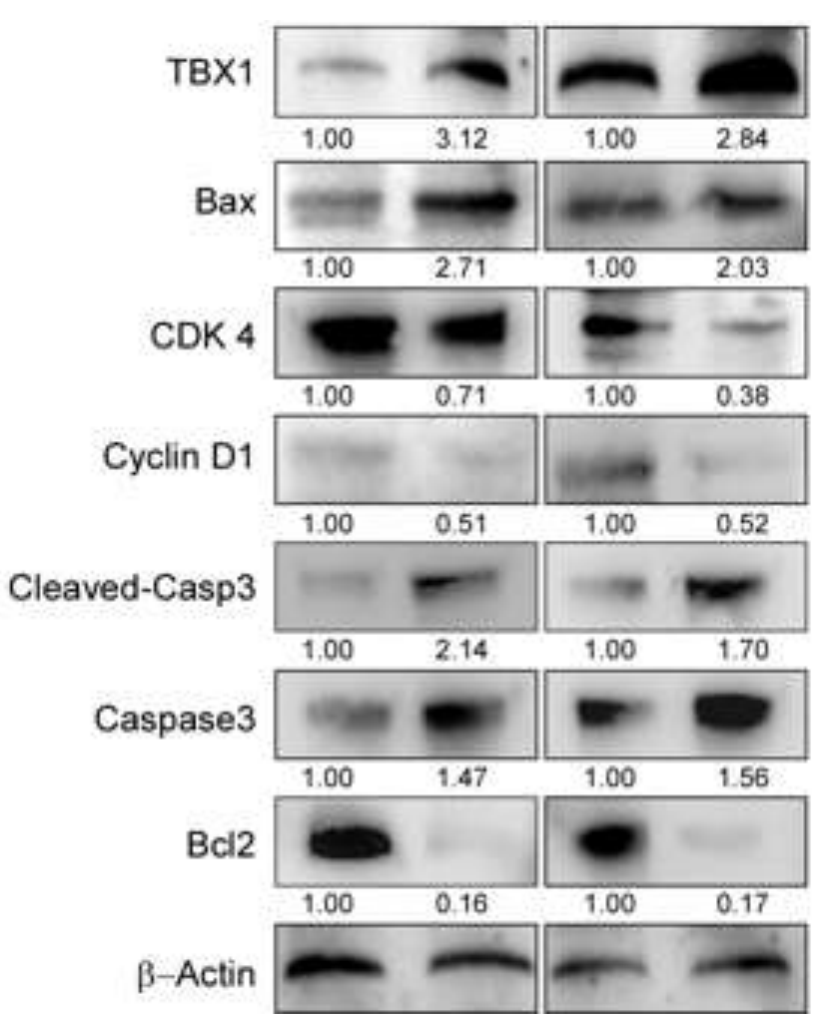

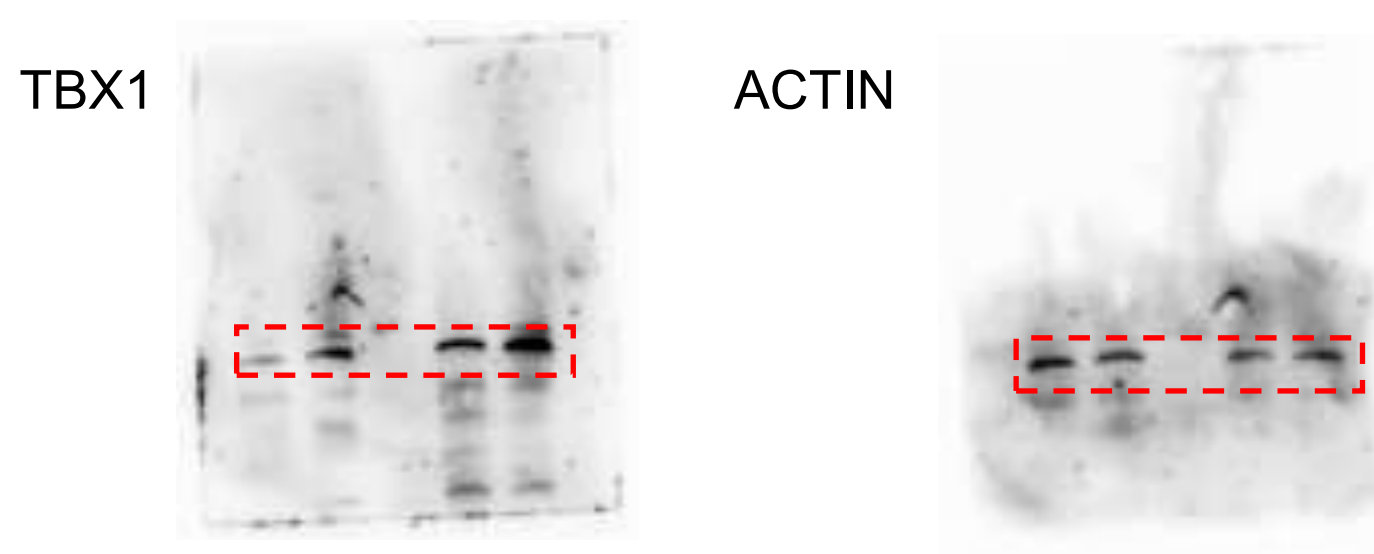

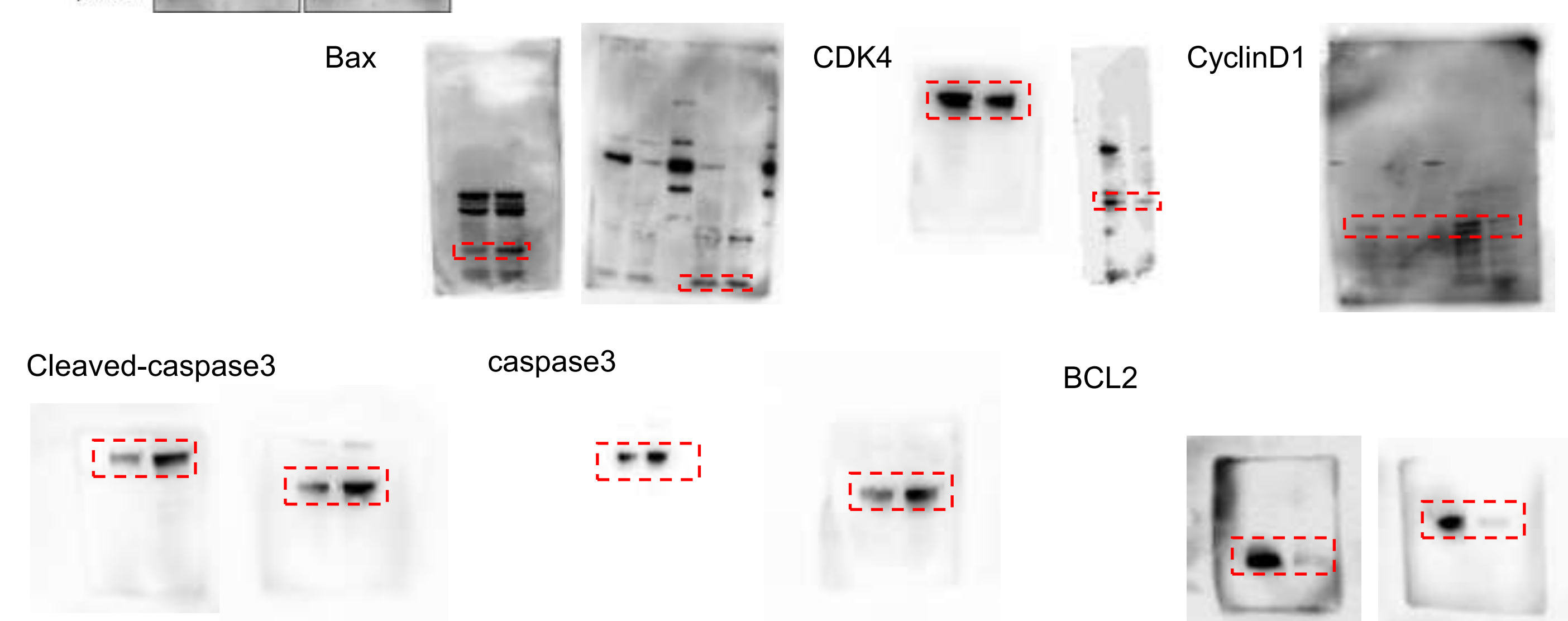

Full unedited gel for Figure 4B

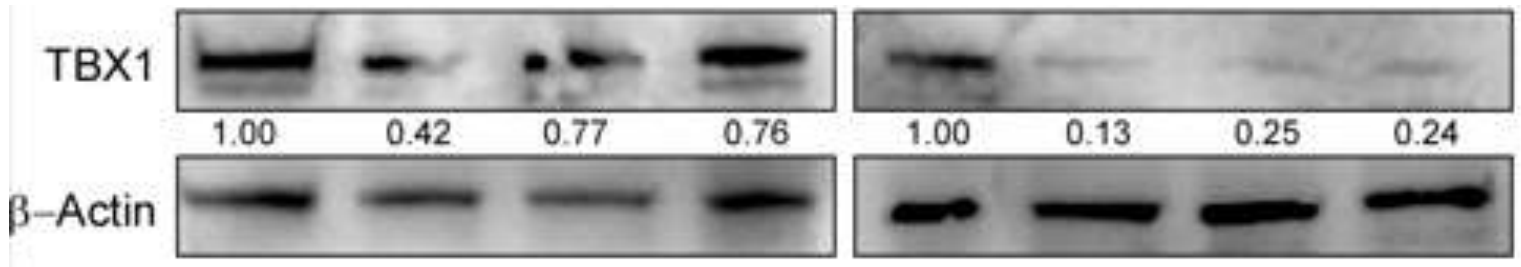

TBX1

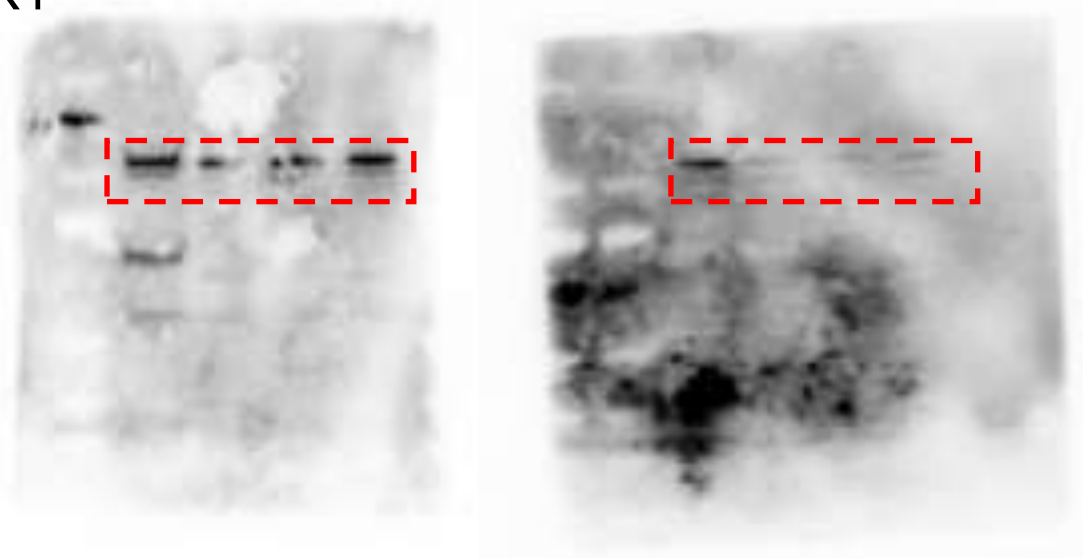

ACTIN

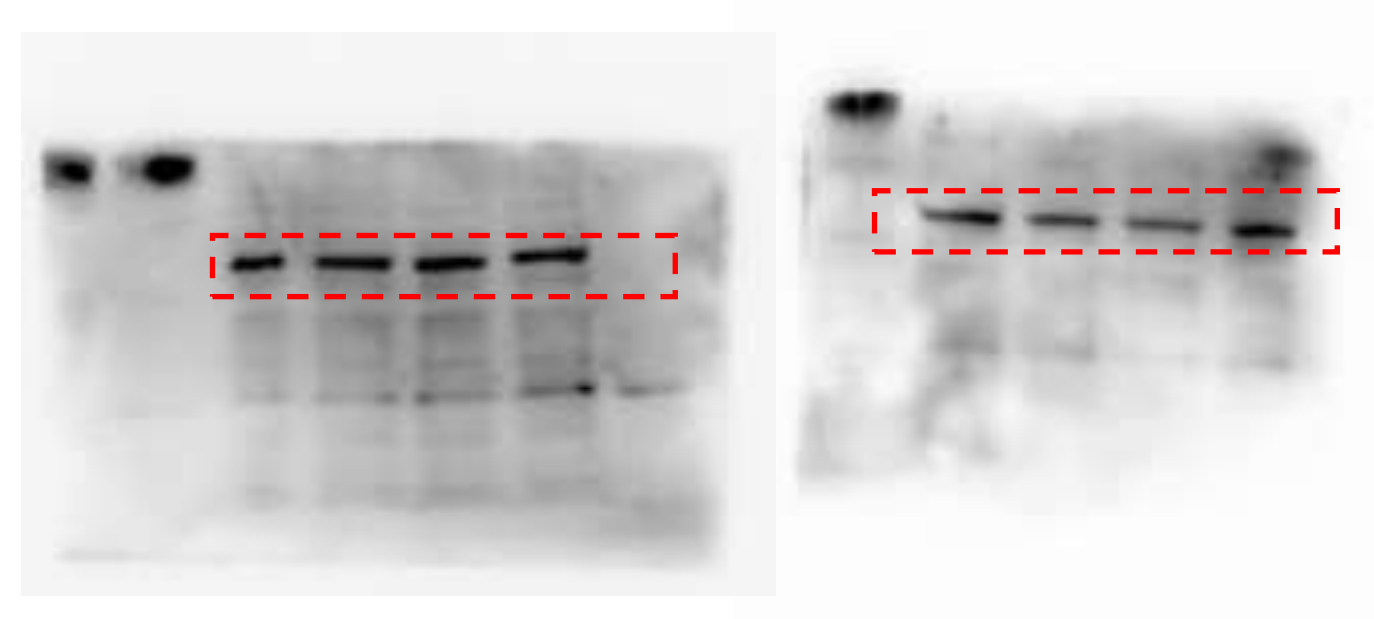

Full unedited gel for Figure 4F

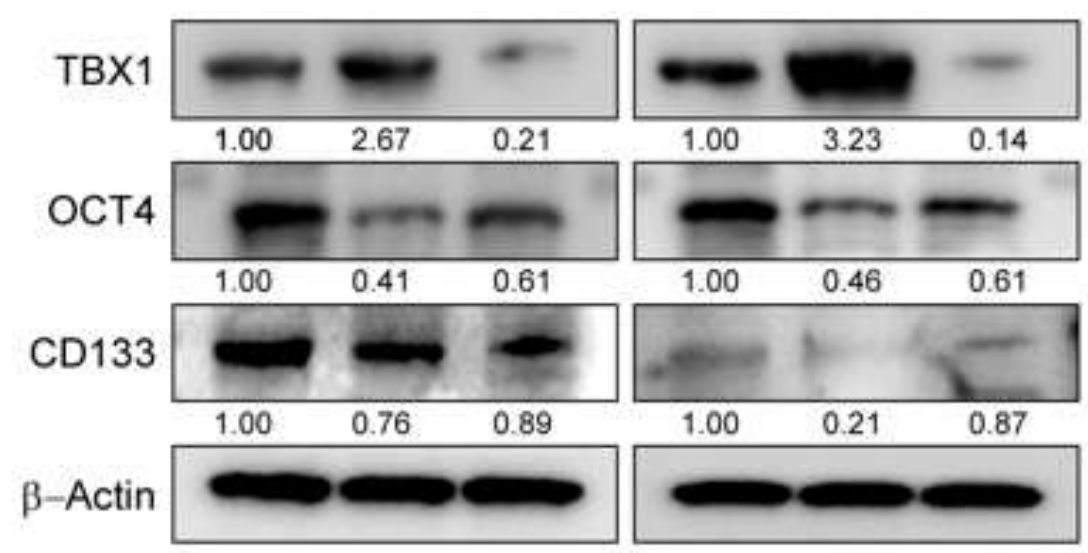

TBX1

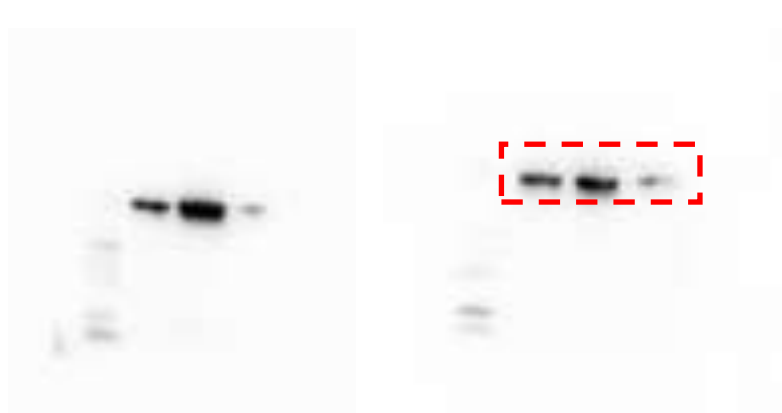

OCT4

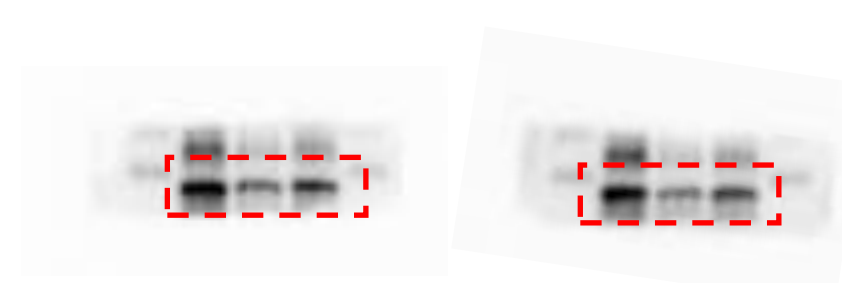

CD133

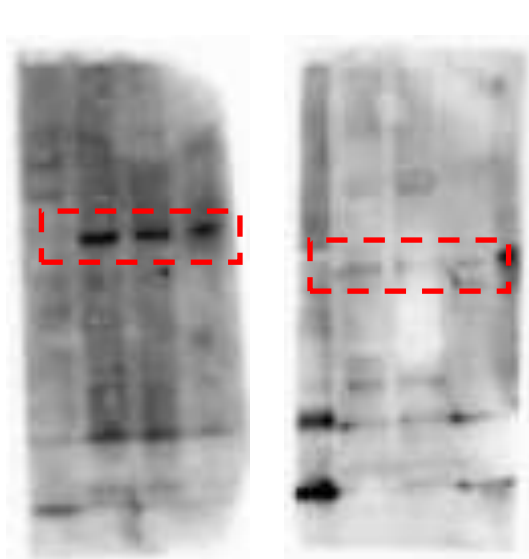

ACTIN

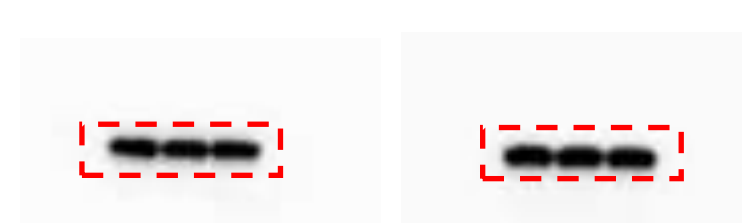

Full unedited gel for Figure 6K

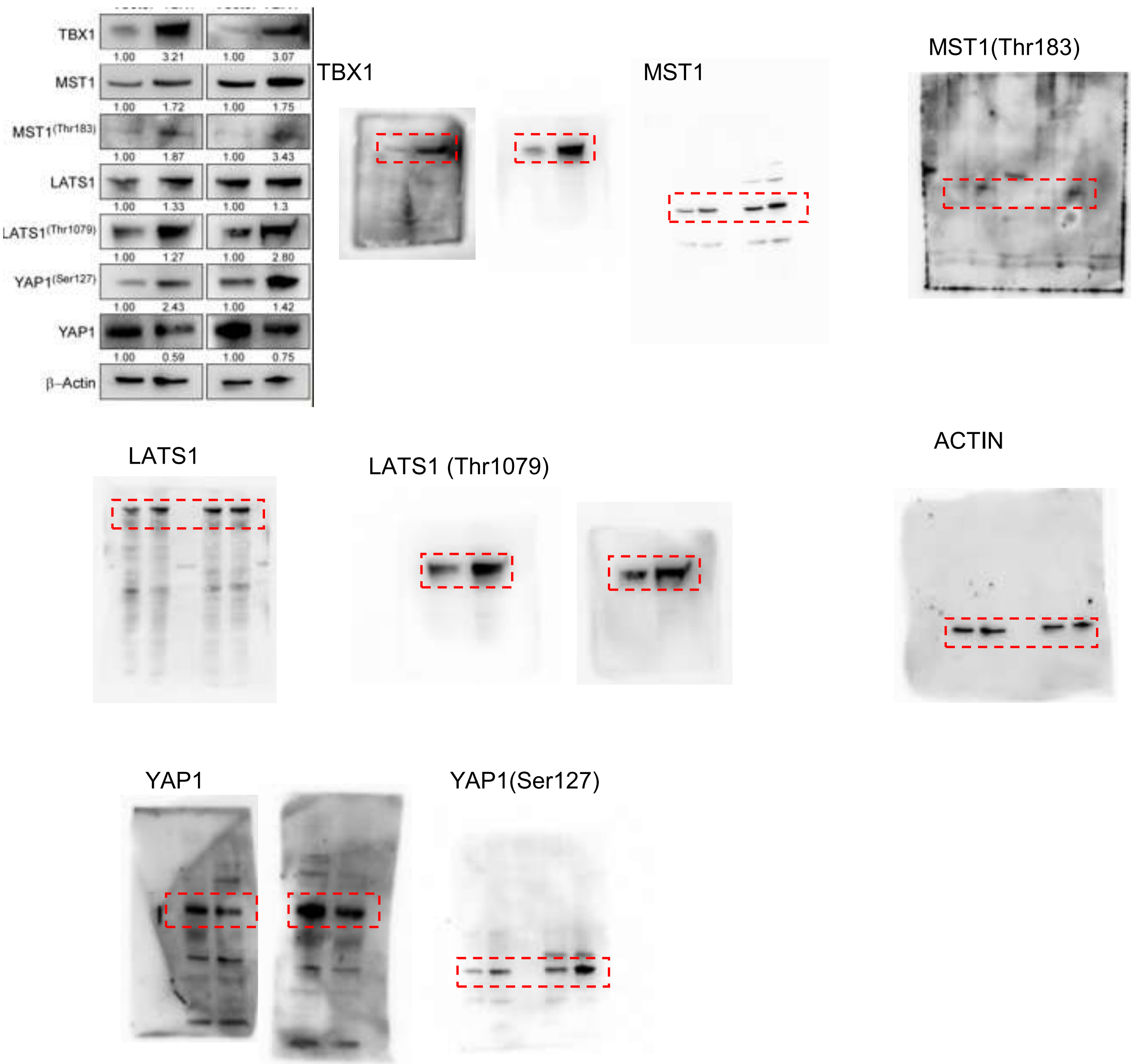

Full unedited gel for Figure 7G

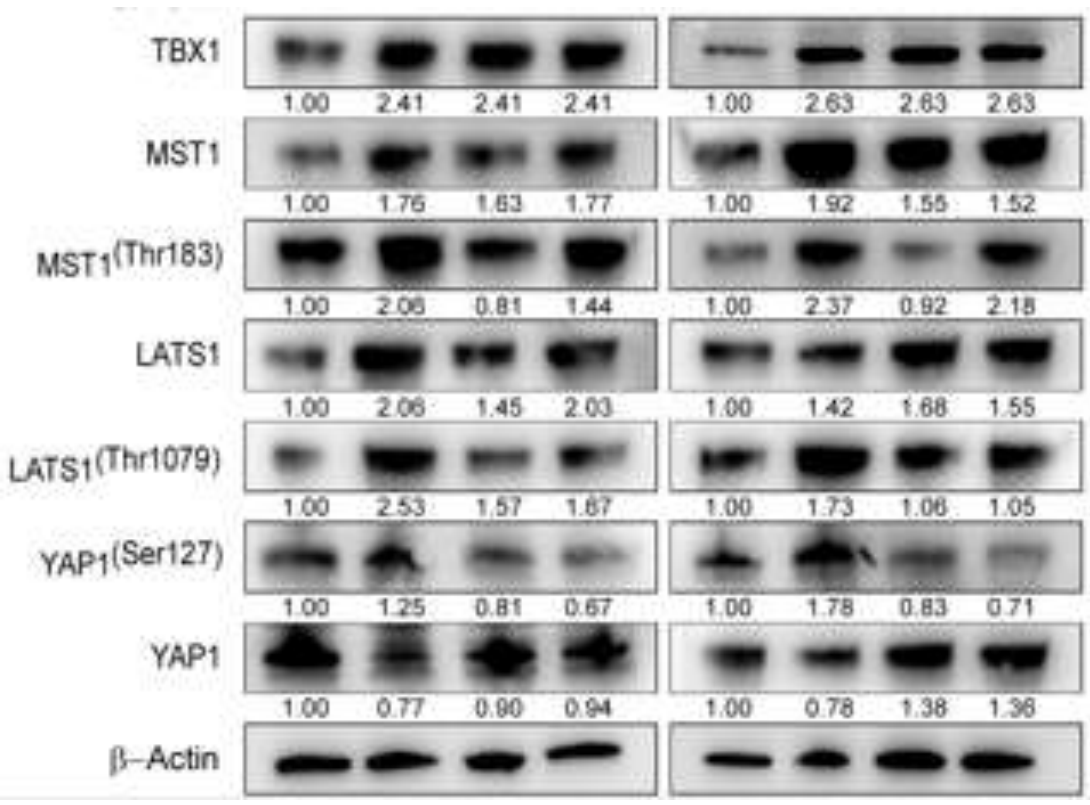

TBX1

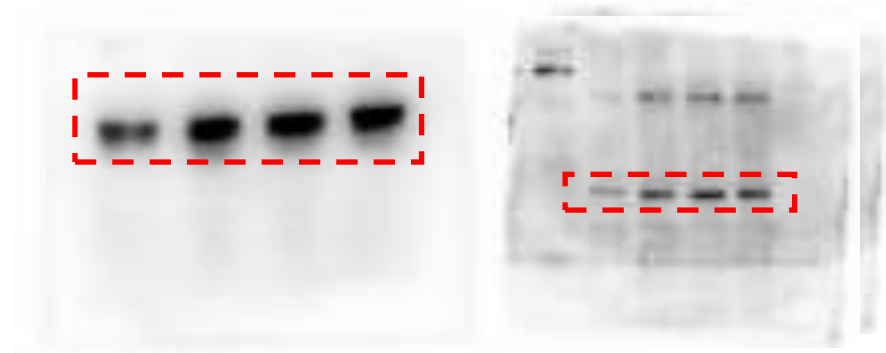

MST1

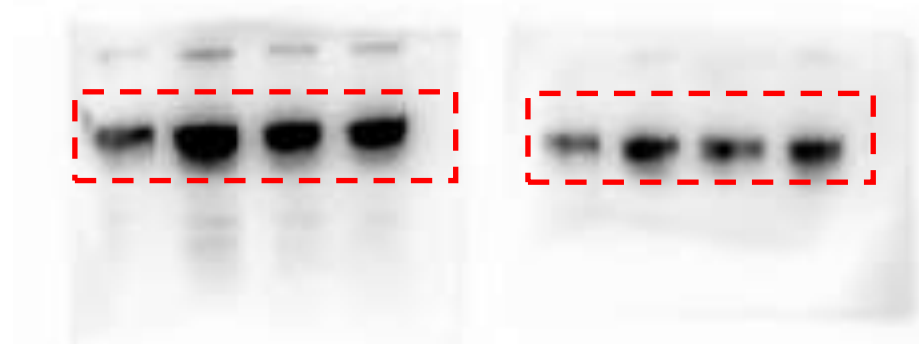

MST1(Thr183)

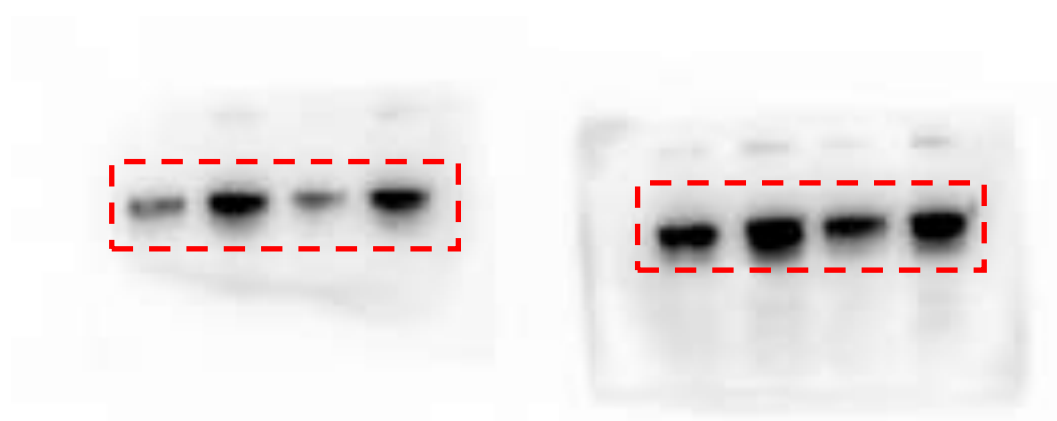

LATS1

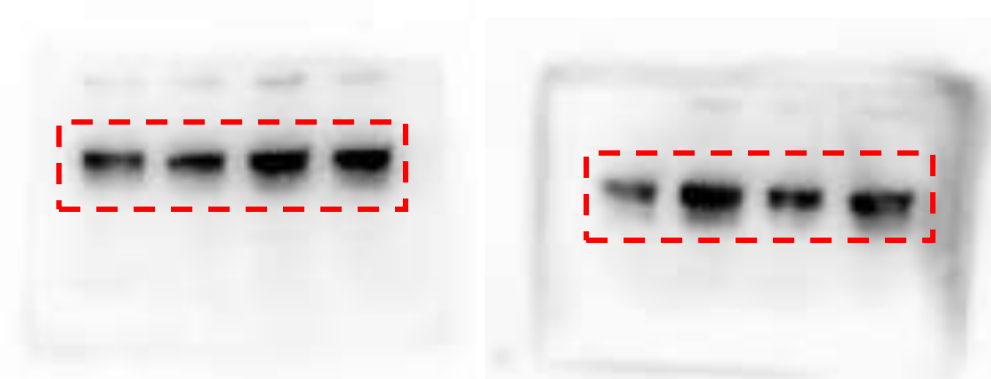

LATS1(Thr1079)

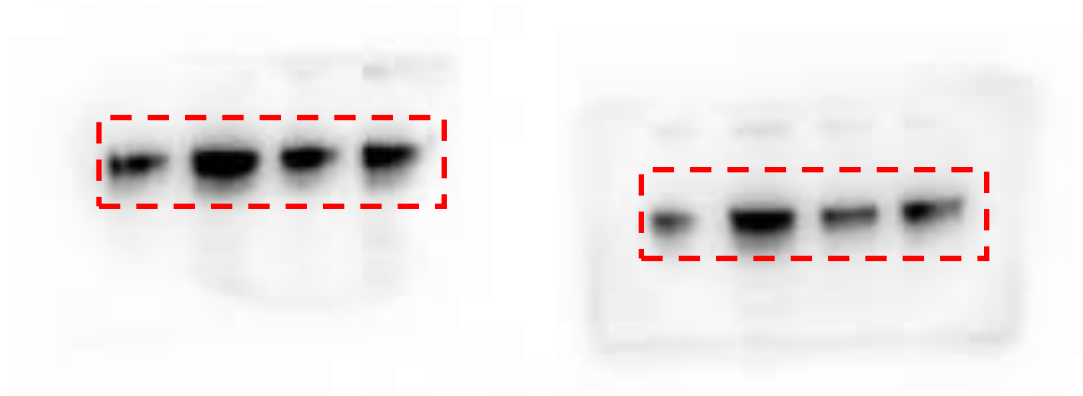

YAP1(Ser127)

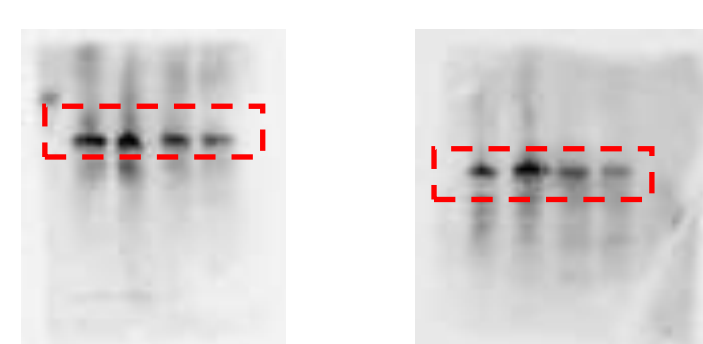

YAP1

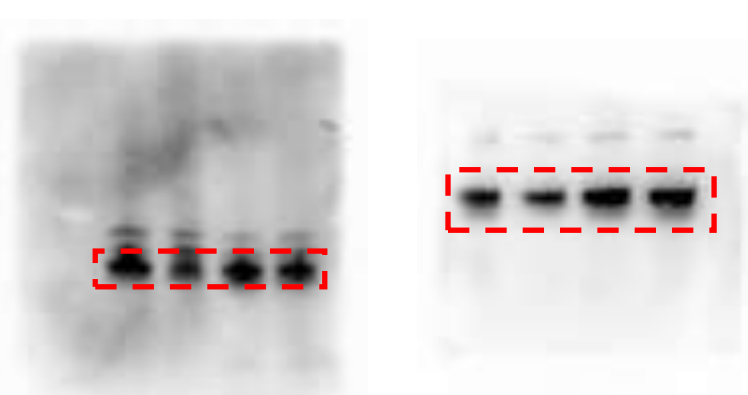

ACTIN

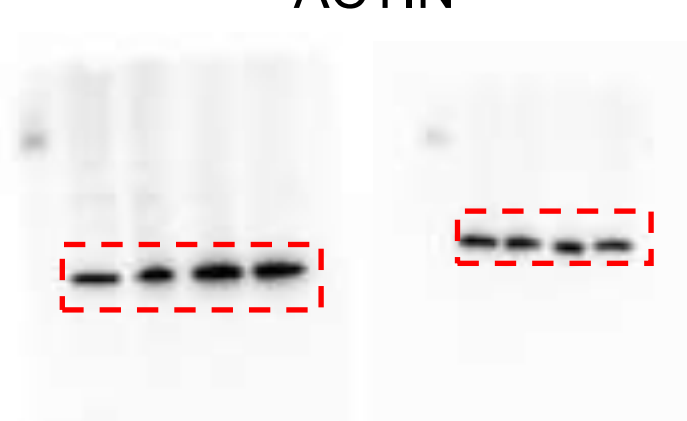

Supplement: Supplementary file 1 [file 41419_2026_8934_MOESM2_ESM.pdf]
